# An explanation of the number of peaks and symmetries of starbursts

**DOI:** 10.1038/s41598-026-53243-7

**Published:** 2026-06-21

**Authors:** Sergio Barbero, Antonia María Delgado, Lidia Fernández

**Affiliations:** 1 Instituto de Óptica, CSIC, Madrid, Spain; 2 Instituto de Matemáticas IMAG and Departamento de Matemática Aplicada, Universidad de Granada, Granada, Spain

**Keywords:** Starbursts, Starbursts symmetries, Zernike polynomials symmetries, Catastrophe optics, Optics and photonics, Physics

## Abstract

Starbursts are the light-intensity patterns seen when small bright sources are observed at low illumination levels, typically stars at night. Starburst patterns are formed because the eye’s wave aberrations generate caustics at the retina. However, a fascinating yet unexplained fact about starbursts is that they usually exhibit *p*-fold symmetry. Moreover, the number of peaks, related to the symmetry perceived by the subject, is not always the same. The main aim of this study is to explain these visual optics phenomena. For this purpose, we provide a theoretical framework based on the geometric and algebraic properties of the wave aberration function expressed as a Zernike polynomial expansion. Specifically, we investigated the number and distribution of the fertile cusps of Gauss of the wave aberration function. We also established the connections between these points with the symmetries and the number of starburst peaks. We found that starbursts are likely generated by wave aberrations dominated by axially symmetric polynomials combined with a certain amount of non-axially symmetric ones. For instance, whereas a wave aberration with a dominant spherical aberration (Zernike polynomial $Z_4^{0}$) plus $Z_3^{3}$ may induce a 3-peaks starburst with a 3-fold symmetry, a wave aberration combining $Z_4^{0}$ and $Z_4^{4}$ may induce a 4-fold symmetry starburst with four or eight peaks. In addition to providing a comprehensive explanation of starburst symmetries, our theory has other promising applications; for instance, we could infer some basic properties of an eye’s wave aberration function from a measurement (subjective or objective) of the starburst pattern.

## Introduction

When looking at a star, under low-light conditions and skies free from excessive artificial light emissions^1^, most people perceive some structured bright patterns, which have been called *starbursts*. Nowadays, it is recognized that the origin of starbursts relates to caustic formation at the retina^2,3^.

Optically, a star in the firmament can be modeled (geometrical optics framework) as a point light source from which a line congruence, *i.e.*, a family of rays with an associated orthogonal surface (the *wavefront*), emerges. After refraction within the eye, this wavefront focuses on the retina, generating a caustic pattern instead of a perfect focal point because the eye is far from ideal, introducing what is called *optical aberrations*. Caustics can be mathematically defined –though different definitions exist– as the loci of the centers of curvature of the wavefront^4^; in other words, caustics are the spatial locations where two or more rays intersect, hence providing those locations with a higher concentration of light. So far, we have only mentioned geometrical optics, but a full and detailed explanation of such caustic shapes ultimately requires wave optics^2^. Nevertheless, if only the symmetry properties of the caustic patterns are considered, geometrical optics is enough.

The starbursts are only perceived when the eye is affected by a noticeable amount of aberrations^2,3^. These are mathematically described as the *wave aberration function*, defined as the difference between the actual wavefront and the ideal reference spherical wavefront that would eventually concentrate all rays in a single point. Considering that the angular size of a typical star is much smaller than the maximum resolution of the eye, namely, 1 arcmin, the image of a star at the retina can only be resolved if the amount of wave aberration generates caustics exceeding that threshold resolution value of 1 arcmin^5^.

Starburst patterns can be quite diverse^3^; still, some typical ones are those in which a bright central area is surrounded by clearly marked intensity spikes, hereafter referred to as: *starburst peaks*. We call these starbursts *star-like*, which is not redundant terminology as there are starbursts that are perceived differently, for instance, as a snowflake pattern^3^.

A striking observation about perceived starbursts is that they usually exhibit a relatively distinct *p*-fold symmetry pattern, *i.e.*, they are invariant after rotation by an angle of *2π /p*. Therefore, the index *p* somehow determines the number of peaks of the starburst. In the paper, we only write starburst to economize language, but we note that we refer to star-like *p*-fold symmetric starbursts. An even more surprising fact is that the *p* index of the *p*-fold symmetry is not always the same, and as a consequence, the number of peaks of the perceived starburst differs among subjects. This is exemplified even in paintings throughout history, where stars are represented with a variety of peaks, though art representation has a strong symbolic character.

Recently, Rubinstein^2^ has provided a theoretical framework to explain several geometrical properties of starbursts, such as the presence of straight lines passing through the central bright spot. Here, we extend Rubinstein’s purposes in trying to explain the geometry of starbursts. Specifically, we provide a theoretical explanation of symmetries and the number of peaks based on the deep relationship between symmetry and its preservation from wavefronts to caustics, and an analysis of certain curvature singular points associated with the wave aberration. For these purposes, instead of using Cartesian Taylor polynomials as in^2^, we use Zernike polynomials because they are naturally more suitable for revealing symmetries, and also are the most common mathematical representation of the human eye’s wave aberration^6^. Our investigations are based on singular analysis of the wave aberration function expressed as a combination of Zernike polynomials. This procedure allows us to establish the connections between the number and symmetry of some relevant points –the so-called fertile cusps of Gauss^7^– with the symmetries and the number of starburst peaks.

First, in Section Basic facts about Zernike polynomials, we briefly present the mathematical properties of Zernike polynomials. Next, in Section Theoretical explanation of the number of peaks and symmetries of starbursts, we provide a theoretical framework to explain the symmetries and number of starburst peaks. For this purpose, we prove a set of propositions concerning symmetries (Section Symmetry properties of startbursts) and necessary relations between Zernike coefficients for the appearance of starburst (Section Relation between Zernike coefficients: necessary conditions for the appearance of starbursts). In the next Section Numerical simulations, we provide numerical computations to develop the theoretical results of the previous section. First, we explain how to compute the singular set of the Hessian determinant function and the caustic pattern; second, we compute maps of these quantities for several archetypal starbursts (Section Archetypical examples of starbursts). Finally, in Section Discussion, we summarize results and discuss their implications.

## Basic facts about Zernike polynomials

Zernike polynomials form a complete orthogonal set over the unit disk^8^. They were first introduced by Frits Zernike to analyze images obtained by his phase-contrast microscopy method, for which he received the Nobel Prize^9^. It soon was recognized that these polynomials were especially suitable for optical science, in general, because in most optical systems, the wave aberration is defined over a circular domain: the pupil of the system. Moreover, while Zernike polynomials are not the only orthogonal polynomials over the unit disk (see^10^, Section 2), they provide a strong match to classical optical aberrations, such as defocus, spherical aberration, coma, astigmatism, etc. Naturally, Zernike polynomials are defined in polar coordinates (*ρ*, *θ*), with *ρ* normalized to unity. These coordinates can be converted to Cartesian coordinates, for which a convention is required. Particularly, a direction and a reference axis for *θ* must be chosen^8^; in this paper, we selected the y-axis as the reference axis and a clockwise direction as in references^11,12^. Zernike polynomials are constructed as products of univariate functions, one depending on *ρ* and the other on *θ*, and some normalization factors^8^:

$$\begin{aligned} \begin{aligned}&Z_{n}^{m}(\rho , \theta ) = N_{n}^{m} R_{n}^{m} (\rho ) \cos (m\theta ), \\&Z_{n}^{-m}(\rho , \theta ) = N_{n}^{m} R_{n}^{m} (\rho ) \sin (m\theta ), \end{aligned} \end{aligned}$$

for $0\le m \le n$ and *n-m* an even number. The normalization factors take the values $N_{n}^{m}=\sqrt{2(n+1)}$ if *m≠ 0* and $N_{n}^{m}=\sqrt{n+1}$ if *m=0*, and the functions $R_{n}^{m}$ are polynomials of degree *n* in the variable *ρ*, and they are defined by a power series:

$$\begin{aligned} R_{n}^{m}(\rho ) = \sum _{i=0}^{(n-m)/2} \frac{(-1)^i(n-i)!}{i!\left( (n+m)/2-i\right) !\left( (n-m)/2-i \right) !}\rho ^{n-2i}, \qquad n \ge m\ge 0, \ \ n-m \text { even}. \end{aligned}$$

These polynomials are related to the classical Jacobi polynomials with the standard normalization^13^ as

$$\begin{aligned} R_{n}^{m}(\rho ) = (-1)^{(n-m)/2}\rho ^m P_{(n-m)/2}^{(m,0)}(1-2\rho ^2), \qquad n \ge m\ge 0, \ \ n-m \text { even}. \end{aligned}$$

With this definition, it holds that $R_{n}^{m}(1)=1$ and they satisfy the following orthogonality conditions:

$$\begin{aligned} \int _0^1 R_n^m(\rho ) R_{n'}^m(\rho ) \rho = \frac{1}{2n+2}\delta _{n,n'}. \end{aligned}$$

Moreover, with a change of variables to Cartesian coordinates $x=\rho \sin (\theta )$, $y=\rho \cos (\theta )$, we have that Zernike polynomials are, indeed, polynomials in *x* and *y*, of total degree *n*, and they are orthonormal on the unit disk $D=\{(x,y)\in \mathbb {R}^2: x^2+y^2\le 1\}$, this is

$$\begin{aligned} \int _{D} Z_n^m(x,y) Z_{n'}^{m'}(x,y) \, dx \, dy = \delta _{n,n'}\, \delta _{m,m'}. \end{aligned}$$

In Zernike polynomials $Z_n^m(\rho ,\theta )$, the parameter *ρ* represents the radial distance from the center of the pupil, normalized so that it ranges from 0 at the center to 1 at the outer edge. The index *n* sets the overall radial order of the polynomial, governing how the function and its curvature vary with radius between the center and the edge. The index *m* sets the angular dependence of the mode: its magnitude specifies how many times the pattern repeats around the circle. Whereas modes with *m=0* are rotationally symmetric, nonzero values introduce angular lobes whose number increases with *m*. Therefore, the indices *n* and *m* describe how the wave aberration function varies both radially and angularly within a circular aperture. Lower values correspond to simple aberrations like defocus or primary astigmatism, while higher ones correspond to higher-order aberrations such as coma, spherical aberration, etc.

**Fig. 1:**
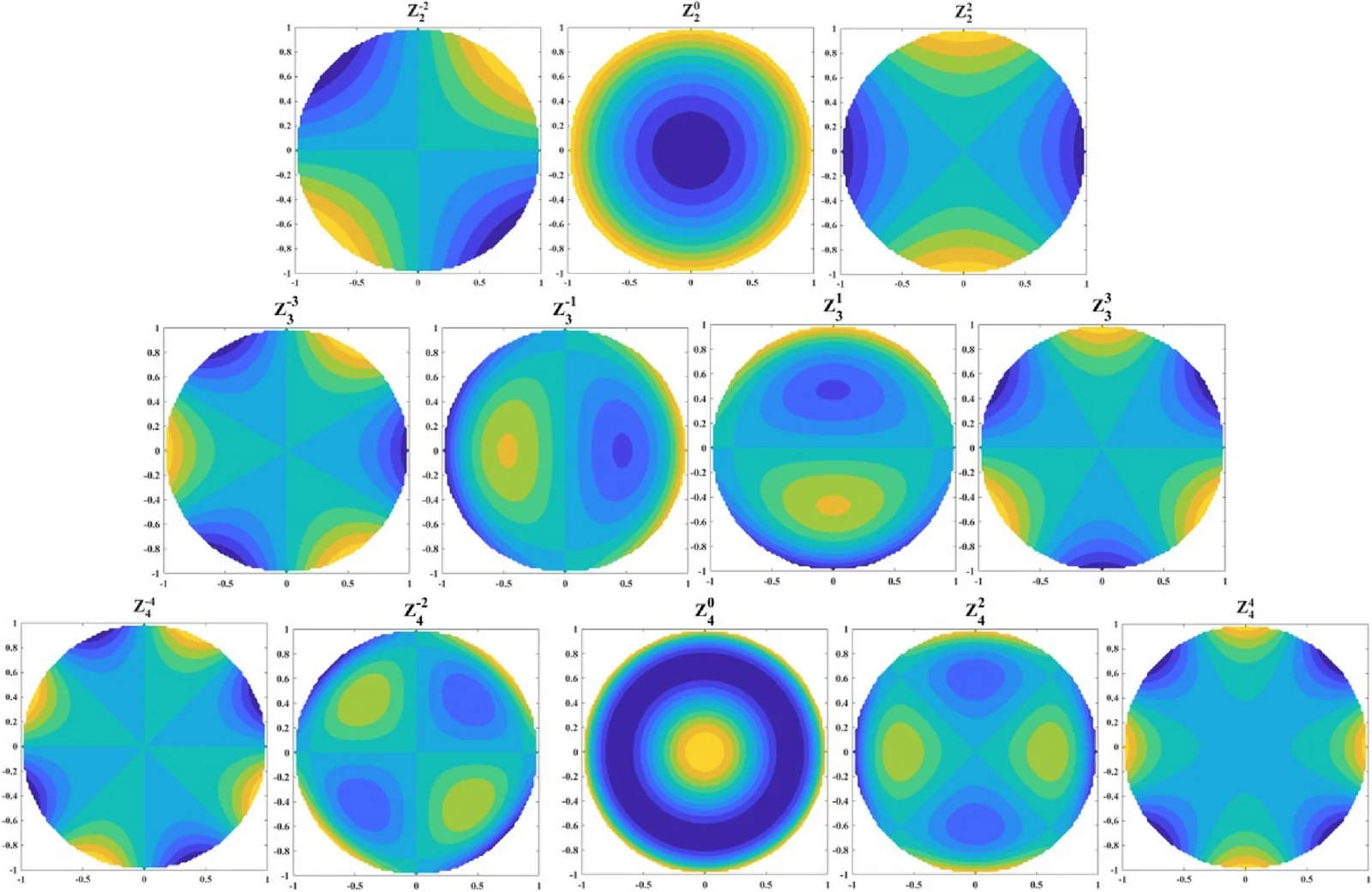
Zernike polynomials up to the fourth degree. $Z_{2}^{0}$ and $Z_{4}^{0}$ corresponds with defocus and spherical aberration, respectively.

Explicit expressions for the first Zernike polynomials are listed in Table 1. Figure 1 represents the normalized Zernike polynomials up to the fourth degree.

**Table 1:** Explicit expression of Zernike polynomials (degrees *n=2,3,4*) in polar and cartesian coordinates and the correspondence with optical aberrations.

*n*	*m*	$Z_n^m(\rho ,\theta )$	$Z_n^m(x,y)$	Aberration
2	*-2*	$\sqrt{6}\rho ^2\sin (2\theta )$	$2\sqrt{6}xy$	Oblique astigmatism
	0	$\sqrt{3} \left( 2 \rho ^2-1\right)$	$\sqrt{3} \left( 2 x^2+2 y^2-1\right)$	Defocus
	2	$\sqrt{6} \rho ^2 \cos (2 \theta )$	$\sqrt{6} \left( y^2-x^2\right)$	Vertical astigmatism
3	*-3*	$\sqrt{8} \rho ^3 \sin (3 \theta )$	$\sqrt{8} x \left( 3 y^2-x^2\right)$	Vertical trefoil
	*-1*	$\sqrt{8} \left( 3 \rho ^3-2 \rho \right) \sin (\theta )$	$\sqrt{8} x \left( 3 x^2+3 y^2-2\right)$	Vertical coma
	1	$\sqrt{8} \left( 3 \rho ^3-2 \rho \right) \cos (\theta )$	$\sqrt{8} y \left( 3 x^2+3 y^2-2\right)$	Horizontal coma
	3	$\sqrt{8} \rho ^3 \cos (3 \theta )$	$\sqrt{8} y \left( y^2-3 x^2\right)$	Oblique trefoil
4	*-4*	$\sqrt{10} \rho ^4 \sin (4 \theta )$	$4 \sqrt{10} x y (y^2-x^2)$	Oblique quadrafoil
	*-2*	$\sqrt{10} \left( 4 \rho ^4-3 \rho ^2\right) \sin (2 \theta )$	$2 \sqrt{10} x y \left( 4 x^2+4 y^2-3\right)$	Oblique secondary astigmatism
	0	$\sqrt{5} \left( 6 \rho ^4-6 \rho ^2+1\right)$	$\sqrt{5} \left( 6 x^4+12 x^2 y^2-6 x^2+6 y^4-6 y^2+1\right)$	Primary spherical
	2	$\sqrt{10} \left( 4 \rho ^4-3 \rho ^2\right) \cos (2 \theta )$	$\sqrt{10} \left( -4 x^4+3 x^2+4 y^4-3 y^2\right)$	Vertical secondary astigmatism
	4	$\sqrt{10} \rho ^4 \cos (4 \theta )$	$\sqrt{10} \left( x^4-6 x^2 y^2+y^4\right)$	Vertical quadrafoil

Symmetry properties of Zernike polynomials are specially relevant for this work. Looking at the trigonometric angular functions of Zernike polynomials, it is straightforward to deduce the 2-fold symmetry of all Zernike polynomials up to a *± 1* constant factor; i.e. $Z_{n}^{m}(\rho , \theta ) = (-1)^m Z_{n}^{m}(\rho , \theta + \pi )$. Besides this, Zernike polynomials, when *m = 0*, are axially symmetric polynomials (invariant under any rotation), and those polynomials obeying *n = m* are *m*-fold symmetric. These last two properties lead us to select certain Zernike polynomials combinations to explain starburst symmetries, as will be described later.

## Theoretical explanation of the number of peaks and symmetries of starbursts

The intersection of caustic surfaces with the retina provides a set (sometimes empty) of points and/or curves. Caustics are associated with points of the wave aberration (*W*) where its Hessian determinant (from now on denoted as *G = det(Hess(W))*) is zero (critical set)^14^. Specifically, caustics are obtained by optically mapping the zero set of *G*; *i.e.*, projecting through ray trajectories pupil plane’s points where *G=0*, onto the retina plane. However, the intensity along caustic curves and neighbouring regions is not uniform.

**Fig. 2:**
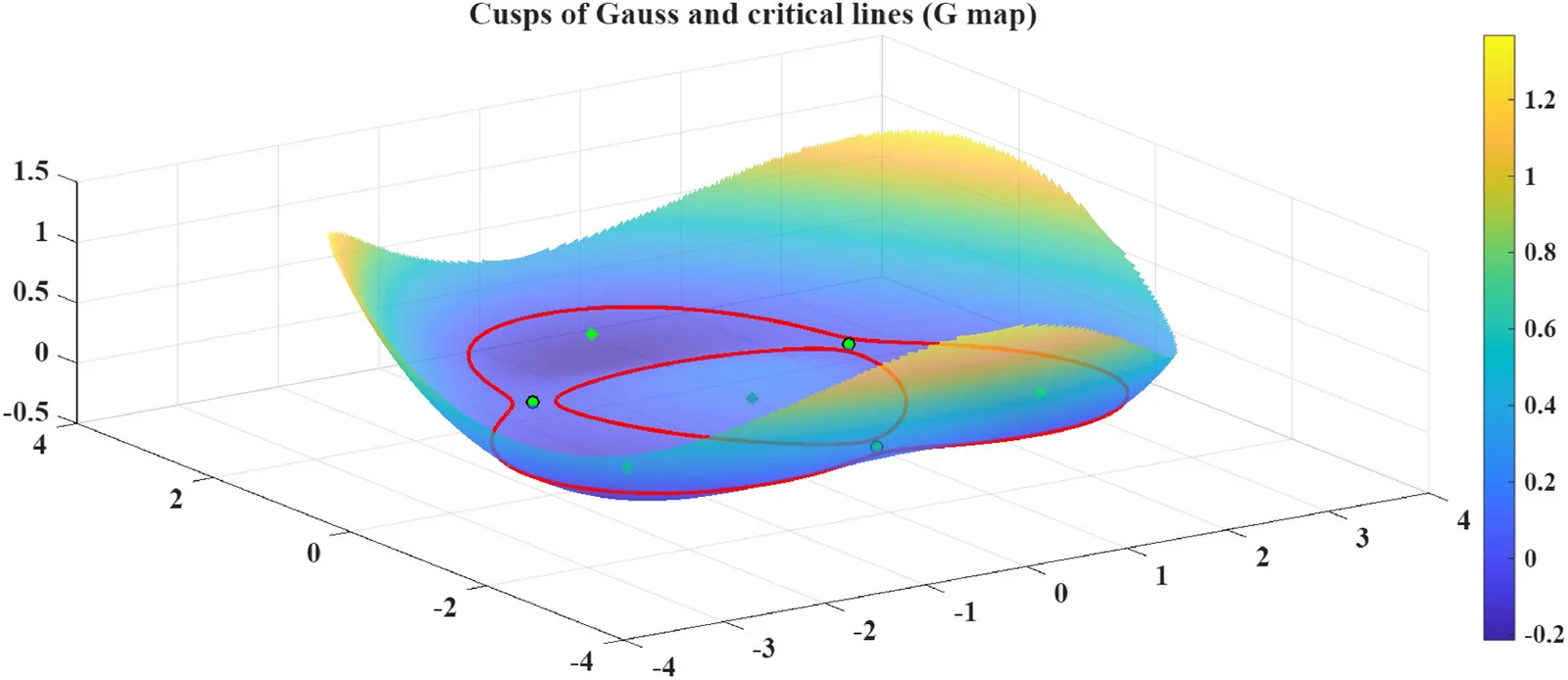
Representation of non-fertile (green stars) and fertile (green points surrounded by black circles) cusps of Gauss and critical lines (red lines) over the G-function of a wave aberration function given by Eq. 6.

Catastrophe optics theory^14–16^ predicts that the presence of stable hot spots of intensity is associated with the existence of some singular wave aberration points called *fertile cusps of Gauss*^7^. These are saddle points of the function *G*, that is, in these points, *∇ G=0* and the Hessian matrix for *G* is indefinite^7^. A matrix is called indefinite if it has both positive and negative eigenvalues, that is, it is not positive semi-definite and not negative semi-definite. This association comes from differential geometry and singular analysis. Cusps in the caustic curves occur when the principal curvature reaches a maximum or a minimum along a line of curvature^15,17^, and these are linked to the aforementioned fertile cusps of Gauss^7^. These points generate hyperbolic catastrophes, giving rise to *G* zero-level contour curves that are close enough to generate hot spots in the intensity^7^. We note that the condition ‘close enough’ is an optical (not mathematical) condition, meaning that sometimes, if the curves separate too much, the hot spot in the intensity is not produced, so the cusp of Gauss is no longer fertile. Figure 2 helps to visualize the difference between a fertile and non-fertile cusp of Gauss. The G-function of the wave aberration given by Eq. 6 is depicted. This function contains 6 cusps of Gauss within the domain. Simple green stars and green points surrounded by black circles represent non-fertile and fertile cusps of Gauss, respectively. Red lines represent the *G* zero-level contour curves. As shown in this example, the fertile, but not the non-fertile, cups of Gauss are close to two *G* zero-level contour curves.

As mentioned before, a necessary condition for a wave aberration function to generate a starburst pattern is that the wavefront concentrates a significant part of the light at the central part of the blurred spot. This is the usual case in most human eyes because wave aberration values are moderately small near the pupil’s center; indeed, if this were not the case, that eye would be pathologically abnormal.

But, given the bright central spot, the starburst peaks are generated due to caustic concentration along them^2,3^. Furthermore, not all the caustics are equally bright; catastrophe theory predicts a much more intense light concentration in the presence of a *cusp* caustic (when the caustic curve contains a discontinuity in the first derivative) or when two *fold* caustics (analytical curves) are close enough. Therefore, we conjecture:

### Hypothesis 1

*Star-like starbursts peaks are perceived only when either a cusp caustic or two very close fold caustics are presented and are separated enough from the central bright spot to be visually resolved*.

These two scenarios imply the existence of fertile cusps of Gauss^7^ generating two branches of *G* zero-level contour curves close to it. When optically mapped onto the retina, these curves may generate a maximum of four-fold and two cusp caustics.

Therefore, the presence of more than one fertile cusp of Gauss, separated from the center area, is mandatory for generating a starburst pattern. In what follows, we will provide a set of mathematical propositions that set the necessary conditions for the appearance and symmetry of some archetypal starburst patterns.

### Symmetry properties of startbursts

Noether’s theorem establishes that every symmetry in the optical Lagrangian of a homocentric beam of rays (all of them sharing a common punctual source) has a corresponding conservation magnitude along its propagation. This law is formalized within variational calculus, and specifically establishes that when the optical Lagrangian has a particular symmetry, meaning that it does not explicitly depend on a given coordinate, its corresponding canonical optical momentum is conserved^18^. Similar conclusions can be derived following the Hamiltonian theory^19^. These arguments support that a link between the symmetries of the wave aberration and the caustic pattern at the retina must exist. We first establish this connection following a different mathematical formalism, which is mostly based on geometric arguments.

A strong connection exists between surface symmetries (in $\mathbb {R}^3$) and their curvature properties. For instance, at a non-umbilic point, the maximum reflection symmetry occurs at planes intersecting orthogonally the surface and/or containing a line of curvature^20^. Moreover, a line embedded in a surface, determining a reflectional surface symmetry, usually contains ridge points (points where the lines of curvature are extremum) (^20^ and ^17^, specifically on p. 271). Cusps of Gauss are associated with ridge points, but with the advantage of being easier to compute^7,17^. Therefore, computing the cusps of Gauss of the wave aberration provides a way to evaluate symmetries. The following proposition provides an important symmetry-preserving property:

#### Proposition 1

*The pattern at the retina plane preserves the p-fold symmetry of the wave aberration at the pupil plane*.

#### Proof

A *p*-fold symmetry implies the presence of *p* planes of symmetric reflection. First, we note that any plane of symmetric reflection of a surface (*S*) intersects it in a geodesic planar curve (*C*). This is because the normal to the surface at any point of a symmetric reflection plane is invariant under reflection, which implies that the normal surface is contained in that plane, and also the normal to a geodesic curve lies in the normal surface by definition. Additionally, every plane geodesic on a surface is also a line of curvature^21^ (specifically on p. 155).

A symmetric reflection plane containing the optical axis is also a meridional plane. Any ray belonging to this meridional plane has zero skewness –part of the vector direction departing from the meridional plane– because it belongs to a geodesic curve^22^, so it remains in that meridional plane through propagation. Therefore, at the retina plane, the curve *C'* imaged by *C* is also a planar curve. Furthermore, the focal curve of a line of curvature is itself a geodesic of the focal surface^17^ (specifically on p. 176). As we have shown above, this implies that it also contains a reflectional planar symmetry. Then, the plane of symmetric reflection in the wave aberration and the caustic surface are the same, which intrinsically implies that the *p*-fold symmetry is preserved between the pupil and retina planes. *□*

The symmetry-preserving property between the wave aberration and the caustic pattern makes it possible, by a simple wave aberration inspection, to realize the *p*-fold symmetry of the starburst pattern and establish a maximum number of starburst peaks. Indeed, this theoretical result agrees with experimental results in which wedge apertures were used to isolate starburst caustic lines^3^.

Zernike polynomial series commonly represents the wave aberration function of the human eye. Recalling that the angular dependent component of a Zernike polynomial is either *sin(m θ )* or *cos(m θ )*, the *p*-fold symmetry of a Zernike polynomial is given by the so-called azimuthal frequency: *m*. The axial symmetry occurs for terms with *m=0*. The symmetries are unclear when several Zernike polynomials combine. Then, the cusps of Gauss emerge as a valuable analytical tool for obtaining geodesic-reflectional symmetric lines. The number of cusps of Gauss imposes an upper bound on the *p*-fold symmetry and, as a consequence, the number of peaks of starbursts, as stated by the following proposition:

#### Proposition 2

*The maximum number of saddle cusps of Gauss is equal to* *(n-2)(2n-5)**, being* *n* *the degree of the wave aberration function* *W, expressed as a linear combination of Zernike polynomials*.

#### Proof

If a surface described by a bivariate polynomial has only isolated critical points, then the number of isolated extrema is bounded above. Also, this bound depends upon the degree of the variables of the polynomial^23^. Let’s say *d* is the degree of a bivariate polynomial containing only isolated critical points. Then, the number of saddle points is bounded above by ${\frac{1}{2} d(d - 1)}$^24^ (specifically on eq. (3.1)).

Now, let us consider *W* the wave aberration function, expressed as a linear combination of Zernike polynomials, with *n* its degree. Thus,

$$G=\det (\textrm{Hess}(W))= W_{xx} W_{yy}-W_{xy}^2,$$

where $W_{xx}$, $W_{yy}$ and $W_{xy}$ represent the second order partial derivatives of *W*. They have degree *≤ n-2*. Then, the degree of *G* is: $d=\deg (G) \le 2(n-2)$. Therefore, the number of saddle points of *G* is, at most, ${\frac{1}{2} d(d - 1)\le }(n-2)(2n-5)$. *□*

With what has been said so far, we analyze which combinations of aberrations give rise to starbursts with different numbers of peaks. The existence of a central intensity core suggests that the wave aberration must contain a combination of axially symmetric terms (defocus plus different orders of spherical aberration) plus some non-axially symmetric terms that break the axial symmetry and generate a *p*-fold symmetry.

We now study several examples of simple combinations of defocus ($Z_{2}^{0}$), spherical aberration ($Z_{4}^{0}$), and dominant *p*-fold symmetric aberrations ($Z_{n}^{n}$, with *n≥ 3*) given rise to starbursts with several peaks. All these combinations are admissible as possible human eye aberrations.

Next, we state that the starburst symmetry pattern is invariant under pupil radius:

#### Proposition 3

*The number and normalized radial locations, concerning the pupil size, of saddle cusps of Gauss are invariant to pupil size*.

#### Proof

This is a direct consequence of the following fact: Let *f*(*x*, *y*) be a bivariate function and define $g(x,y)=f(\frac{x}{r},\frac{y}{r})$. Then it is clear that $\nabla g(rx,ry)=\frac{1}{r}\nabla f(x,y)$ and $\textrm{Hess}\, g(rx,ry)=\frac{1}{r^2}\textrm{Hess}\,f(x,y)$. Therefore, $(x_0,y_0)$ is a saddle point of *f* if, and only if, $(rx_0,ry_0)$ is a saddle point of *g*. *□*

From now on, we are going to consider a wave aberration function expressed as:

1 $$\begin{aligned} W=C_{2,0}\, Z_2^0+C_{4,0}\, Z_4^0 +C_{n,n}\, Z_n^n, \end{aligned}$$

where we use the normalization for Zernike polynomials given in^12^. $C_{2,0}$ and $C_{4,0}$ denote the defocus and spherical aberration coefficients, respectively. $C_{n,n}$ provides the non-axially symmetric component of the wave aberration. We note that the presence of a non-axial dominant $Z_{n}^{n}$ aberration does not imply that *p*-fold symmetry could not appear with other Zernike combinations, but, at least, this is the simplest combination giving rise to that symmetry. Also, we will use, in certain cases, the spherical equivalent, which, as a gross approximation, can be obtained from^25^: $M = \frac{4\sqrt{3}\,C_{2,0}}{Rp^2}$, being $R_p$ the pupil radius. In this formula, $C_{2,0}$ is given in *μ*m and $R_p$ in mm.

#### Proposition 4

*Consider a wave aberration (*1*). If there is a saddle cusp of Gauss at a distance* *ρ>0* *from the center of the pupil, then there are* *n* *saddle cusps of Gauss uniformly distributed at the same distance from the center*.

The proof of this result can be found in Proof of Proposition A.

#### Proposition 5

*Let us consider a wave aberration function defined as in (* 1 *), with* $n \in \{3, 4, 5, 6\}$ *. Then, the number s of saddle cusps of Gauss inside the pupil is:*

- *s=0* *or* *s=n* *when* *n=3,4*,
- *s=0**,* *s=n* *or* *s=2n* *when* *n=5,6*.

*Moreover, if **s=n**, then the points are uniformly distributed in a circle centered at the origin. Also, if **s=2n**, they are uniformly distributed in two circles of different radius*, *npoints in each*.

#### Proof

Following the proof of Proposition 4 given in Proof of Proposition  A, the values of the radius *ρ* where the saddle points are located are solutions of equations $A(\rho ) \pm B(\rho )= 0$ where *A* and *B* are given by (13) and (14), respectively. We distinguish for different values of *n*.

**Case** *n=3*. In this case, both equations $A(\rho ) \pm B(\rho )= 0$ simplify to two quadratic equations in *ρ*:

$$180 \,C_{4,0} ^2 \rho ^2 \pm 9 \sqrt{10} \,C_{4,0} \,C_{3,3} \rho + 4 \,C_{4,0} \Big (\sqrt{15} \,C_{2,0} -15 \,C_{4,0} \Big )-3 \,C_{3,3} ^2 =0,$$

which we can solve explicitly and obtain the four solutions

$$\rho = \frac{\pm 3 \,C_{3,3} \pm \sqrt{32 \,C_{4,0} \left( 15 \,C_{4,0} -\sqrt{15} \,C_{2,0} \right) +33 C_{3,3} ^2}}{12 \sqrt{10} \,C_{4,0} }.$$

Observe here that, if they are real, there are two positive values and two negative ones. To give conditions for these values to be real, *0<ρ <1*, and the corresponding critical points to be saddle cusps of Gauss, we need to define the following parameters:

2 $$\begin{aligned} \begin{aligned}&\alpha _1^\pm =\frac{-120 \,C_{4,0} ^2\pm 9 \sqrt{10} \,C_{4,0} \,C_{3,3} +3 \,C_{3,3} ^2}{4 \sqrt{15} \,C_{4,0}}, \quad \alpha _2=\frac{60 \,C_{4,0} ^2+3 \,C_{3,3} ^2}{4 \sqrt{15} \,C_{4,0}}, \quad \alpha _3=\frac{480 \,C_{4,0} ^2+33 \,C_{3,3} ^2}{32 \sqrt{15} \,C_{4,0}}. \end{aligned} \end{aligned}$$

**Fig. 3:**
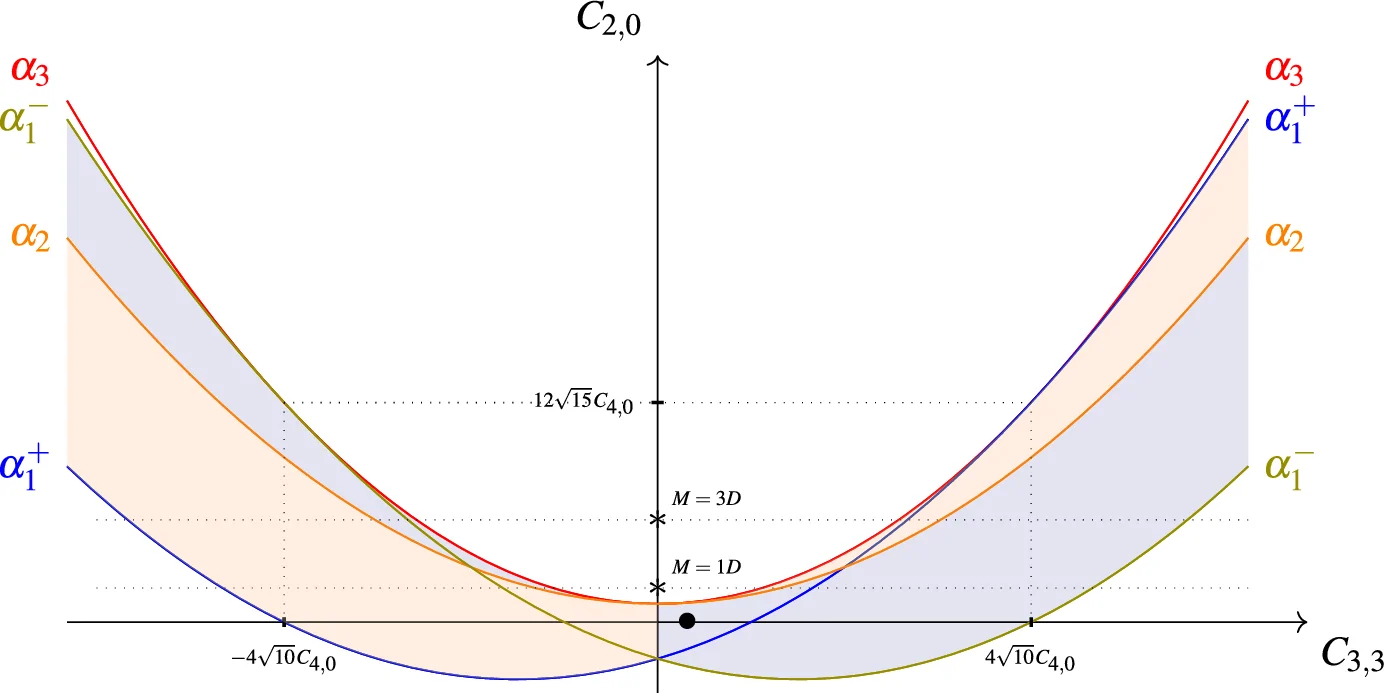
Regions of values for $C_{2,0}$ and $C_{3,3}$ (depending on $C_{0,4}$) providing saddle cusps of Gauss inside the pupil (case *n=3*). Blue areas correspond with saddle points with angular coordinates $\theta =\pi /3,\pi ,5\pi /3$. Orange areas correspond with saddle points with angular coordinates $\theta =0,2\pi /3,4\pi /3$. The black bullet corresponds to the values of example (6). Expressions for $\alpha _1^\pm ,\alpha _2,\alpha _3$ are given in (2). For reference, we indicate in the vertical axis some values for the spherical equivalent $M=\frac{4\sqrt{3}\,C_{2,0}}{R_p^2}$ for a pupil radius of $R_p=3mm$ and a standard value of $C_{4,0}=0.18$.

After some calculations (done using *Mathematica*) we have the following conditions (see Fig. 3):

- there are saddle cusps of Gauss inside the pupil, $(\rho ,\theta )$ with angular values $\theta =0,2\pi /3,4\pi /3$, when

**Table:** 

$C_{3,3}<0$ $\alpha _1^+<C_{2,0}<\alpha _2$	$0<C_{3,3}<4\sqrt{10}\,C_{4,0}$ $\alpha _2<C_{2,0}<\alpha _3$	$C_{3,3}>4\sqrt{10}\,C_{4,0}$ $\alpha _2<C_{2,0}<\alpha _1^+$

- there are saddle cusps of Gauss inside the pupil, $(\rho ,\theta )$ with angular values $\theta =\pi /3,\pi ,5\pi /3$, when

**Table:** 

$C_{3,3}>0$ $\alpha _1^-<C_{2,0}<\alpha _2$	$-4\sqrt{10}\,C_{4,0}<C_{3,3}<0$ $\alpha _2<C_{2,0}<\alpha _3$	$C_{3,3}<-4\sqrt{10}\,C_{4,0}$ $\alpha _2<C_{2,0}<\alpha _1^-$

**Case** *n=4*. In this case, we proceed similarly to the previous one. Equations $A(\rho ) \pm B(\rho )=0$ become

$$\begin{aligned} \begin{aligned} 2 \, C_{4,0}(\sqrt{15} \, C_{2,0} -15\, C_{4,0}) +15 \rho ^2(6\, C_{4,0}^2-C_{4,4}^2 \pm 2 \sqrt{2} \,C_{4,0} \, C_{4,4} ) =0, \end{aligned} \end{aligned}$$

which we can easily solve explicitly, obtaining two positive solutions, one from each equation:

$$\begin{aligned} \rho =\sqrt{\frac{2}{15}}\sqrt{\frac{C_{4,0}(15 \, C_{4,0}-\sqrt{15} \, C_{2,0})}{6 \,C_{4,0}^2\pm 2\sqrt{2} \, C_{4,0} \, C_{4,4} -C_{4,4}^2}}. \end{aligned}$$

We observe that the solution with the minus sign corresponds with the equation $A(\rho ) - B(\rho )=0$ which comes with the angles $\theta =0,\pi /2,\pi ,3\pi /2$, whereas the solution with the plus sign corresponds with $A(\rho ) + B(\rho )=0$ which comes with the angles $\theta =\pi /4,3\pi /4,5\pi /4,7\pi /4$. Again using *Mathematica*, we explore the conditions to guarantee the existence of saddle cusps of Gauss inside the pupil. Let us define:

3 $$\begin{aligned} \alpha _1^\pm =\frac{-60 \, C_{4,0}^2\pm 30\sqrt{2}\, C_{4,0} \, C_{4,4} +15 \, C_{4,4}^2}{2\sqrt{15} \, C_{4,0}}. \end{aligned}$$

**Fig. 4:**
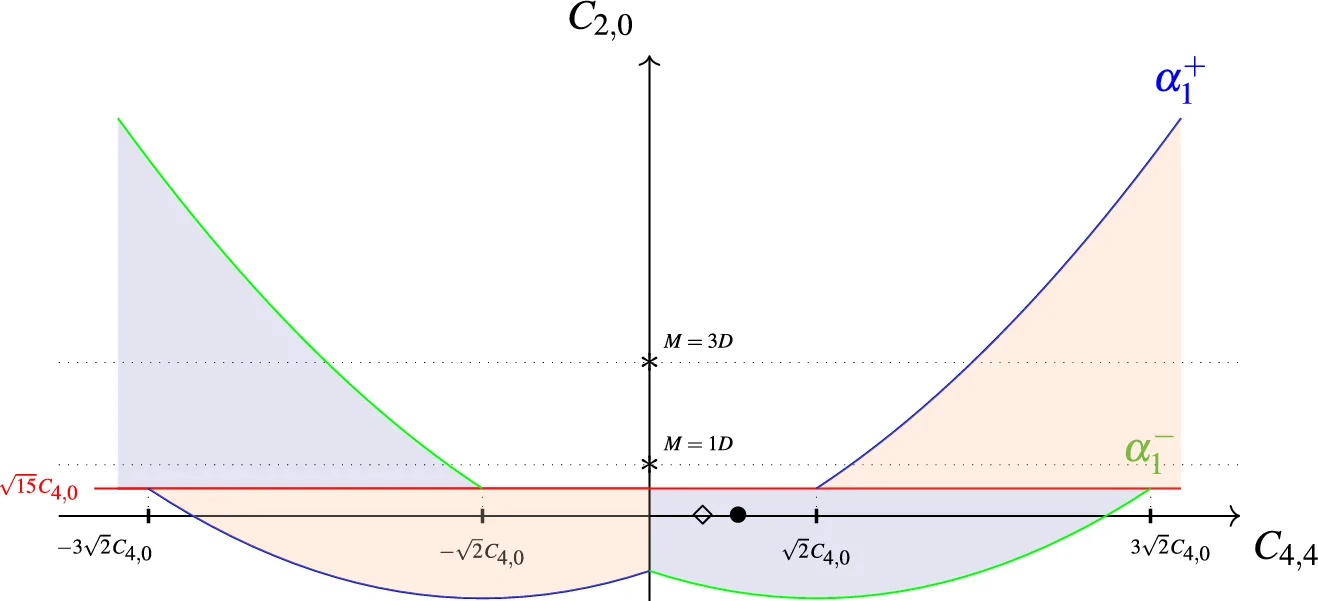
Regions of values for $C_{2,0}$ and $C_{4,4}$ (depending on $C_{4,0}$) providing saddle cusps of Gauss inside the pupil (case *n=4*). Blue areas correspond with saddle points with $\theta =\pi /4,3\pi /4,5\pi /4,7\pi /4$. Orange areas correspond with saddle points with angular coordinates $\theta =0,\pi /2,\pi ,3\pi /2$. Black bullets correspond with the election of values, for example (8), and diamonds with example (10). Expressions for $\alpha _1^\pm$ are given in (3). For reference, we indicate in the vertical axis some values for the spherical equivalent $M=\frac{4\sqrt{3}\,C_{2,0}}{R_p^2}$ for a pupil radius of $R_p=3mm$ and a standard value of $C_{4,0}=0.18$.

Then, there are saddle cusps of Gauss inside the pupil, $(\rho ,\theta )$ (see Fig. 4):

- with $$\theta =0,\pi /2,\pi ,3\pi /2$$, when

**Table:** 

$-3\sqrt{2} \, C_{4,0}<C_{4,4}<0$ $\alpha _1^+<C_{2,0}<\displaystyle \sqrt{15} \, C_{4,0}$	$C_{4,4}>\sqrt{2} \, C_{4,0}$ $\displaystyle \sqrt{15} \, C_{4,0}<C_{2,0}<\alpha _1^+$

- with $$\theta =\pi /4,3\pi /4,5\pi /4,7\pi /4$$ when

**Table:** 

$0<C_{4,4}<3\sqrt{2} \, C_{4,0}$ $\alpha _1^-<C_{2,0}<\displaystyle \sqrt{15 }\,C_{4,0}$	$C_{4,4}<-\sqrt{2} \, C_{4,0}$ $\displaystyle \sqrt{15} \, C_{4,0}<C_{2,0}<\alpha _1^-$

**Case** *n=5*. In this case, we proceed again as previously. Equations $A(\rho ) \pm B(\rho )=0$ become

$$2 \sqrt{15} \, C_{4,0}(C_{2,0} -\sqrt{15} \, C_{4,0} ) +90 \, C_{4,0} ^2 \, \rho ^2\pm 25 \sqrt{15}\, C_{4,0} \, C_{5,5} \, \rho ^3-75\, C_{5,5} ^2 \, \rho ^4 = 0,$$

whose solutions can be explicitly solved with *M*athematica. In the discussion, we will need some parameters that we define here

4 $$\begin{aligned} \begin{aligned}&\alpha _1^\pm =\frac{-60 \, C_{4,0}^2\pm 25\sqrt{15} \, C_{4,0} \, C_{5,5} +75 \, C_{5,5}^2}{2\sqrt{15}\,C_{4,0}} , \quad \alpha _2^\pm =\frac{9 \left( 4561\pm 445 \sqrt{89}\right) \,C_{4,0}^3}{1024 \sqrt{15}\,C_{5,5} ^2}, \quad \gamma _1^\pm = \frac{\sqrt{15} \left( \sqrt{89}\pm 5\right) \,C_{4,0}}{40} . \end{aligned} \end{aligned}$$

**Fig. 5:**
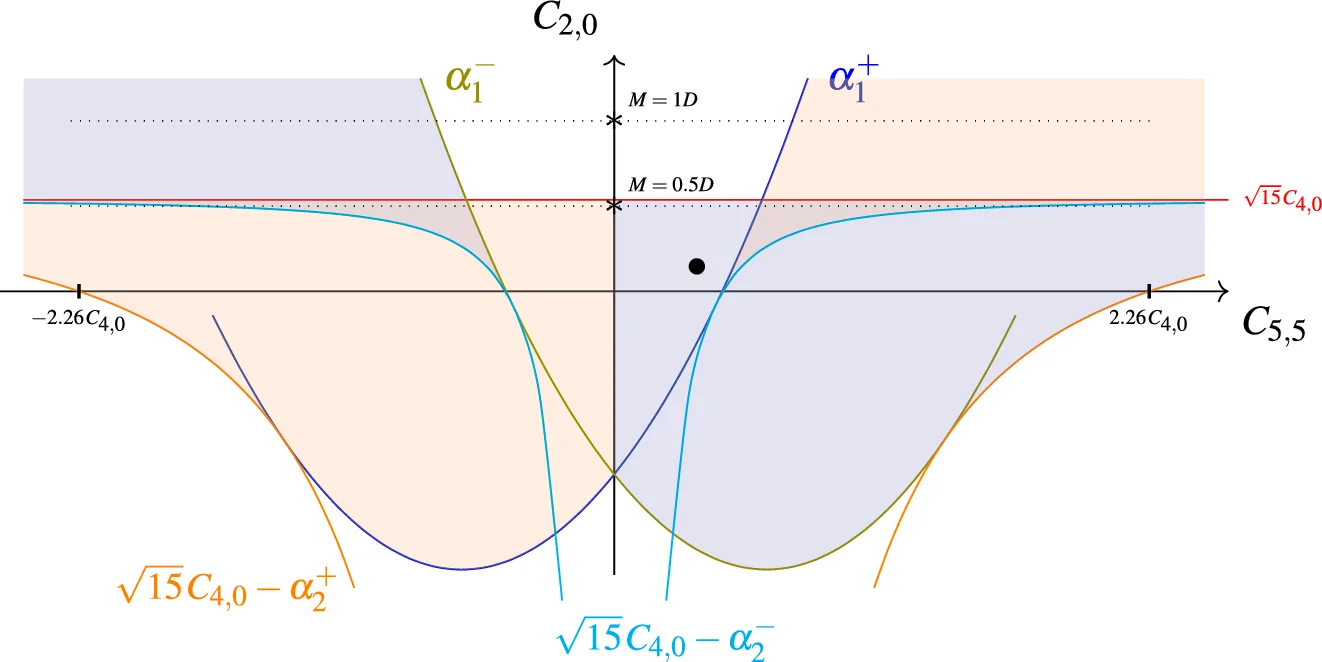
Regions of values for $C_{2,0}$ and $C_{5,5}$ (depending on $C_{4,0}$) providing saddle cusps of Gauss inside the pupil (case *n=5*). Blue areas correspond with saddle points with angular coordinates $\theta =\pi /5,3\pi /5,\pi ,7\pi /5,9\pi /5$. Orange areas correspond with saddle points with $\theta =0,2\pi /5,4\pi /5,6\pi /5,8\pi /5$. The black bullet corresponds with values of example (7). Expressions for $\alpha _1^\pm , \alpha _2^\pm , \gamma _1^\pm$ are given in (4). For reference, we indicate in the vertical axis some values for the spherical equivalent $M=\frac{4\sqrt{3}\,C_{2,0}}{R_p^2}$ for a pupil radius of $R_p=3mm$ and a standard value of $C_{4,0}=0.18$.

Then, there are saddle cusps of Gauss inside the pupil, $(\rho ,\theta )$ in the following conditions (see Fig. 5):

- with $$\theta =\frac{\pi }{5},\frac{3\pi }{5},\pi ,\frac{7\pi }{5},\frac{9\pi }{5}$$, if

**Table:** 

$C_{5,5}<-\gamma _1^-$ $\sqrt{15}\,C_{4,0}-\alpha _2^-<C_{2,0}<\alpha _1^+$	$0<C_{5,5}<\gamma _1^+$ $\alpha _1^-<C_{2,0}<\sqrt{15}\,C_{4,0}$	$C_{5,5}>\gamma _1^+$ $\sqrt{15}\,C_{4,0}-\alpha _2^+<C_{2,0}<\sqrt{15}\,C_{4,0}$

- with $$\theta =0,\frac{2\pi }{5},\frac{4\pi }{5},\frac{6\pi }{5},\frac{8\pi }{5}$$, if

**Table:** 

$C_{5,5}<-\gamma _1^+$ $\sqrt{15}\,C_{4,0}-\alpha _2^+<C_{2,0}<\sqrt{15}\,C_{4,0}$	$-\gamma _1^+<C_{5,5}<0$ $\alpha _1^+<C_{2,0}<\sqrt{15}\,C_{4,0}$	$C_{5,5}>\gamma _1^-$ $\sqrt{15}\,C_{4,0}-\alpha _2^-<C_{2,0}<\alpha _1^+$

In Fig. 5, we can see the different regions. We observe that there are two pieces with saddle cusps of Gauss located at two radii with different angles, namely:

**Table:** 

$C_{5,5}>\gamma _1^-$ $\sqrt{15}\,C_{4,0}-\alpha _2^-<C_{2,0}<\min \{\alpha _1^+,\sqrt{15}\,C_{4,0}\}$	$C_{5,5}<-\gamma _1^-$ $\sqrt{15}\,C_{4,0}-\alpha _2^-<C_{2,0}<\min \{\alpha _1^-,\sqrt{15}\,C_{4,0}\}$

**Case** *n=6*. In this case, equations $A(\rho ) \pm B(\rho )=0$ simplify to

$$4\sqrt{15}(C_{2,0}-\sqrt{15}\,C_{4,0})\,C_{4,0} + 180\,C_{4,0}^2\rho ^2 \pm 45\sqrt{70}\,C_{4,0}\,C_{6,6}\,\rho ^4 - 525\,C_{6,6}^2\,\rho ^6 = 0,$$

which is a six-degree polynomial equation with only even degrees. Then, we can make $t=\rho ^2$ and get cubic equations in *t*, which we can explicitly solve.

Let us define some parameters that will help us to describe the conditions under which saddle cusps of Gauss appear.

5 $$\begin{aligned} \begin{aligned}&\alpha _1^\pm =\frac{-120\,C_{4,0}^2\pm 45\sqrt{70}\, C_{4,0}\,C_{6,6} +525\,C_{6,6}^2}{4\sqrt{15}\,C_{4,0}}, \quad \alpha _2^\pm = \frac{C_{4,0}^2}{C_{6,6}} \sqrt{\frac{2}{7}}\left( 9\pm 4\sqrt{3}\right) , \quad \gamma _1^\pm =C_{4,0}\frac{\sqrt{210}\pm \sqrt{70}}{35}. \end{aligned} \end{aligned}$$

**Fig. 6:**
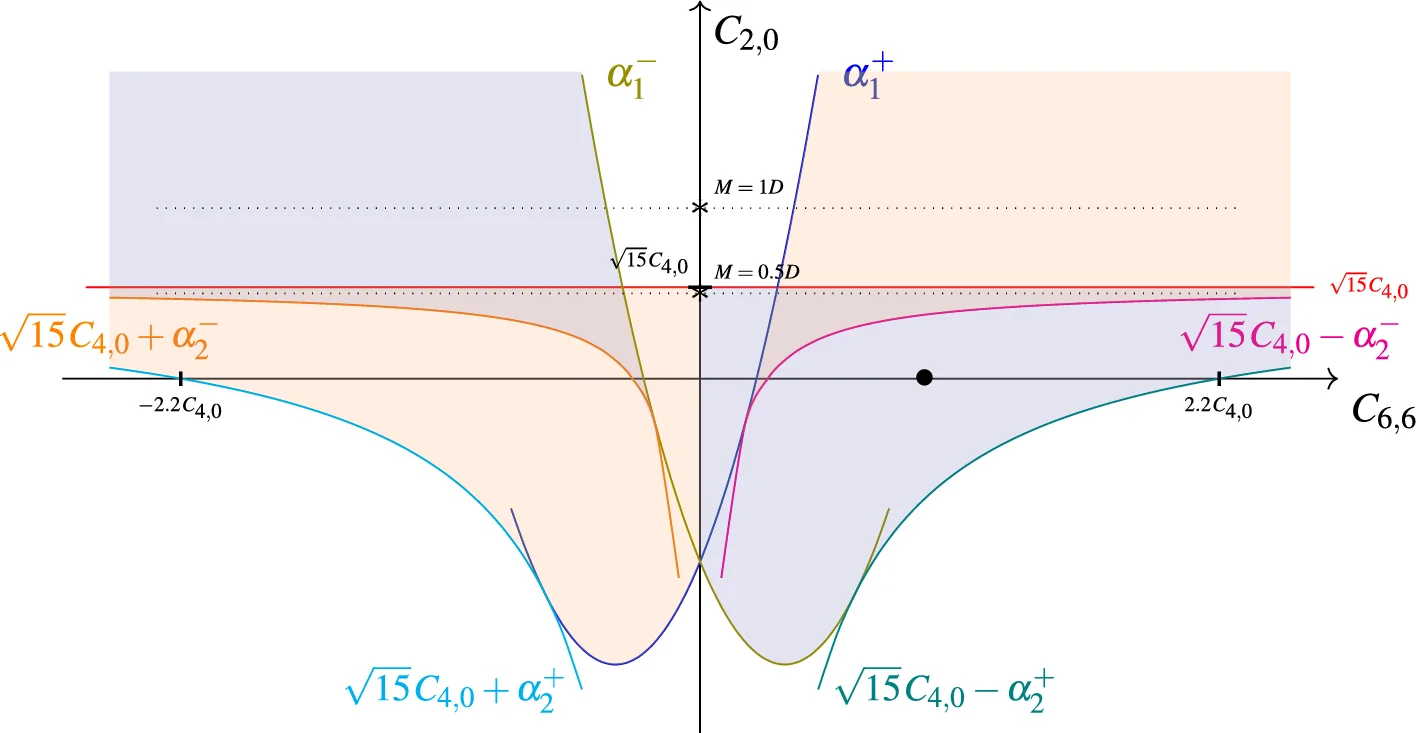
Regions of values for $C_{2,0}$ and $C_{6,6}$ (depending on $C_{4,0}$) providing saddle cusps of Gauss (case *n=6*). Blue areas correspond with saddle points with $\theta =\pi /6,3\pi /6,5\pi /6,7\pi /6,9\pi /6,11\pi /6$. Orange areas correspond with saddle points with $\theta =0,2\pi /6,4\pi /6,\pi ,8\pi /6,10\pi /6$. The black bullet corresponds with the values of example (9). Expressions for $\alpha _1^\pm , \alpha _2^\pm , \gamma _1^\pm$ are given in (5). For reference, we indicate in the vertical axis some values for the spherical equivalent $M=\frac{4\sqrt{3}\,C_{2,0}}{R_p^2}$ for a pupil radius of $R_p=3mm$ and a standard value of $C_{4,0}=0.18$.

Doing computations with *Mathematica*, we get that there are saddle cusps of Gauss inside the pupil, $(\rho ,\theta )$, in the following situations (see Fig. 6):

- with $$\theta =\frac{\pi }{6},\frac{3\pi }{6},\frac{5\pi }{6},\frac{7\pi }{6},\frac{9\pi }{6},\frac{11\pi }{6}$$ if:

**Table:** 

$C_{6,6}<-\gamma _1^-$ $\sqrt{15}\,C_{4,0} + \alpha _2^-<C_{2,0}<\alpha _1^-$	$0<C_{6,6}<\gamma _1^+$ $\alpha _1^-<C_{2,0}<\sqrt{15}\,C_{4,0}$	$C_{6,6}\ge \gamma _1^+$ $\sqrt{15}\,C_{4,0}-\alpha _2^+<C_{2,0}<\sqrt{15}\,C_{4,0}$

- with $$\theta =0,\frac{2\pi }{6},\frac{4\pi }{6},\pi ,\frac{8\pi }{6},\frac{10\pi }{6}$$ if

**Table:** 

$C_{6,6}>\gamma _1^+$ $\sqrt{15}\,C_{4,0} - \alpha _2^-<C_{2,0}<\alpha _1^+$	$-\gamma _1^+<C_{6,6}<0$ $\alpha _1^+<C_{2,0}<\sqrt{15}\,C_{4,0}$	$C_{6,6}\le -\gamma _1^+$ $\sqrt{15}\,C_{4,0}+\alpha _2^+<C_{2,0}<\sqrt{15}\,C_{4,0}$

Here we have to observe that there are two regions where we can get twelve saddle points within two different circles, these regions are

**Table:** 

$C_{6,6}<-\gamma _1^+$ $\sqrt{15}\,C_{4,0}+\alpha _2^-<C_{2,0}<\min \{\sqrt{15}\,C_{4,0} , \alpha _1^-\}$	$C_{6,6}>\gamma _1^-$ $\sqrt{15}\,C_{4,0}-\alpha _2^-<C_{2,0}<\min \{\sqrt{15}\,C_{4,0} , \alpha _1^+\}$

*□*

As a consequence, following Proposition 1 and Proposition 5, we can state that *n* determines the maximum *p*-fold symmetry of the star pattern.

#### Corollary 6

*A p-fold symmetric wave aberration may only generate a starburst that either has p peaks which are uniformly distributed in a circle centered at the pupil center, or it has 2p peaks, where p lay in a circle, and also the other p peaks, but at a different distance (say shorter), with the condition that each of the long-short pairs of starburst peaks are aligned along the same symmetric reflection plane*.

#### Proof

In each meridian plane, being a plane of symmetry, there are only a maximum of two starburst peaks. If there is only one, due to *p*-fold symmetry, there can only be *p* starburst peaks. In the other case, if each plane of symmetry contains two starburst peaks, the same structure must be replicated in the rest of the planes of symmetry to maintain the *p*-fold symmetry, so there are 2*p* starburst peaks. *□*

Finally, another important implication of the relations between $C_{2,0}$, $C_{4,0}$, and $C_{n,n}$, for all the studied cases illustrated in Figs. 1 to 4, is that the admissible values of defocus ($C_{2,0}$ value) for the appearance of saddle cusps of Gauss are bounded. In other words, if the subject looking at a star has a certain level of defocus (for instance, because not correcting its refraction with spectacles), he or she could not see the star-like starburst. We also depicted, in Figs. 3-6, some reference values of spherical equivalent *M* to illustrate this relevant fact.

### Relation between Zernike coefficients: necessary conditions for the appearance of starbursts

Under low-light conditions (required to see starbursts), a typical value of pupil radius is 3.5 *mm*; we use this value from now on. Some statistical studies on the distribution of Zernike aberrations in the normal population and healthy eyes have revealed that higher-order aberrations tend to be randomly distributed about a mean value of zero^25^, except for spherical aberration. Also, there is no apparent correlation between the degree of ametropia and spherical aberration^26^. The mean value of spherical aberration ($Z_{4}^{0}$ accompanying coefficient in the Zernike expansion of the wave aberration function) is positive and around $0.18\,\mu m$ over a *3 mm* pupil radius (*Rp*)^26^. Taking into account these reference values, in (1) we set $C_{4,0} = 0.2\,\mu m$ using a *3.5 mm* pupil radius. The coefficients accompanying the Zernike terms are given in *μ m*.

Considering the previous observations, we analyze only limited values –still wide enough to capture the totality of possible real eye cases– of the parameters for defocus ($C_{2,0}$ as the coefficient of $Z_{2}^{0}$), spherical ($C_{4,0}$ as the coefficient of $Z_{4}^{0}$), and $Z_{n}^{n}$ (with coefficient $C_{n,n}$) aberration.

Specifically, we take $C_{4,0}=0.2$, and analyze the situation for values of $C_{n,n}$ in the appropriate interval given in the proof of Proposition 5 for the different values of *n*. In particular, in absence of defocus ($C_{2,0}=0$), the admissible values for $C_{n,n}$ are in the intervals:

- $$C_{3,3}\in [-2.5,2.5]$$ for *n=3*,
- $$C_{4,4}\in [-0.76,0.76]$$ for *n=4*,
- $$C_{5,5}\in [-0.45,0.45]$$ for *n=5*,
- $$C_{6,6}\in [-0.44,0.44]$$ for *n=6*.

## Numerical simulations

In this section, we explain the algorithms used to compute the set of critical curves (*G=0*); cusps of Gauss: *∇ G = 0*; saddle cusp of Gauss: *∇ G = 0* with $\det (\text {Hess}\,G )< 0$, and caustic patterns, and apply them to obtain different maps for several archetypical examples of starbursts.

### Computation of critical and singular points of the Hessian determinant and caustic patterns

We denote (*x*, *y*) the coordinates of a wavefront aberration function (*W*) at the eye’s exit pupil. A ray at this plane is optically propagated onto the retina, assumed as usual to be a plane. However, in visual optics, it is better to provide angular magnitudes at the retina, denoted with $(\xi , \eta )$. These coordinates are: *ξ = x'/f* and *η = y'/f*, where *(x', y')* are the ray Cartesian coordinates at the retina plane, and *f* is the distance between this plane and the exit pupil plane.

The relation between (*x*, *y*) and $(\xi , \eta )$ is given by^7^:

6 $$\begin{aligned} \xi = -\frac{\partial W}{ \partial x}, \quad \eta = -\frac{\partial W}{\partial y}. \end{aligned}$$

We note that here, contrary to what was used in reference^7^, the defocus aberration ($Z_2^0$) is explicitly included in *W*.

From the analytical expression of *W*, we obtain algebraic expressions of *G*, and subsequently numerically evaluate it over a fine Cartesian uniform mesh within the circular domain of the eye exit pupil. To compute the set *G=0*, we first interpolate the discrete set of data of *G*, and later obtain the iso-level curves using Matlab built-in functions *contour* and *getContourLineCoordinates*.

To compute the cusps of Gauss, we first numerically solve, using Matlab built-in function *vpasolve*, the set of two equations provided by *∇ G = 0*, and subsequently select those solutions that are saddle: $\det (\text {Hess}\,G )< 0$.

To compute the caustic curves at the retina plane, we optically map the set *G=0* with equations (Computation of critical and singular points of the Hessian determinant and caustic patterns). The same procedure applies to obtaining the cups of Gauss locations projected to the retina plane.

### Archetypical examples of starbursts

As previously mentioned, we concentrate on star-like starbursts, *i.e.*, with a *p*-fold symmetry. A *p*-fold symmetric starburst can be classified depending on the number of peaks. A first classification is based on the parity of the number of peaks, so that we can say an odd or even starburst. A second classification concerns the number of peaks and their length. There are two possibilities: *p* equally-spaced peaks from the starburst center, or 2*p*, where *p* peaks are the same length, and the other *p* peaks have another length; one set is due to cusp caustics and the other to two very close fold caustics (see hypothesis 1). We name the first equally-distanced peaks starbursts and the second non-equally-distanced peaks starbursts. Our methodology allowed us to find four archetypical starbursts: 3, 4, 5, and 6 equally-distanced peaks starbursts, and an 8 non-equally-distanced peaks starburst.

**Fig. 7:**
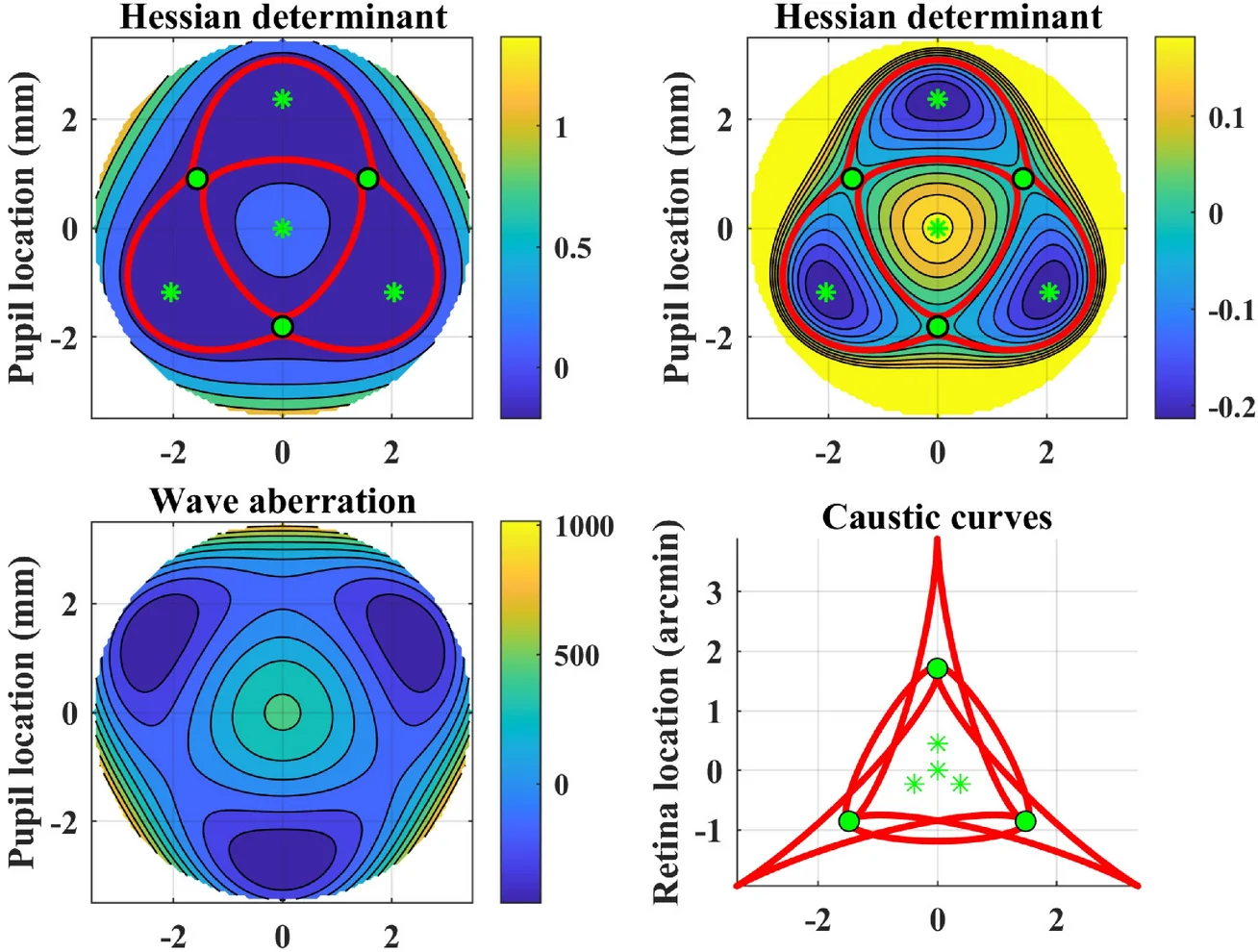
Wave aberration $W = 0.2 Z_{4}^{0} + 0.2 Z_{3}^{3}$ given in (6), Hessian determinant maps, and caustic patterns of equally-distanced three-point starburst. The same Hessian map is depicted in the upper panels. However, in the right panel the colorbar is clipped to specific values around zero to reveal the fine structure of the Hessian determinant in regions located close to the cusps of Gauss. Green stars denote the cusps of Gauss, and green points surrounded by black circles denote the saddle cusps of Gauss in both the Hessian determinant map and its projection onto the retina plane.

#### Odd equally-distanced peaks starbursts

The first example is a wave aberration given by:

6 $$\begin{aligned} W = 0.2 Z_{4}^{0} + 0.2 Z_{3}^{3}. \end{aligned}$$

Figure 7 shows the wave aberration, Hessian determinant of *W*, and caustic pattern generated by (6). We provide two Hessian determinant maps. In the left column, the colorbar is proportional to the Hessian determinant through the pupil; however, in the right column, the colorbar is clipped to specific values around zero to reveal the structure of the Hessian determinant in regions near the cusps of Gauss.

The wave aberration preserves the 3-fold symmetry (Proposition 1) between the Hessian determinant map and the caustic pattern. Overall, there are 7 cusps of Gauss, 3 being fertile (saddle cusps generating near fold caustics), setting the planes of specular symmetry and giving rise to a three-point starburst pattern because of the caustic cusps associated with each of the three fertile cusps of Gauss.

**Fig. 8:**
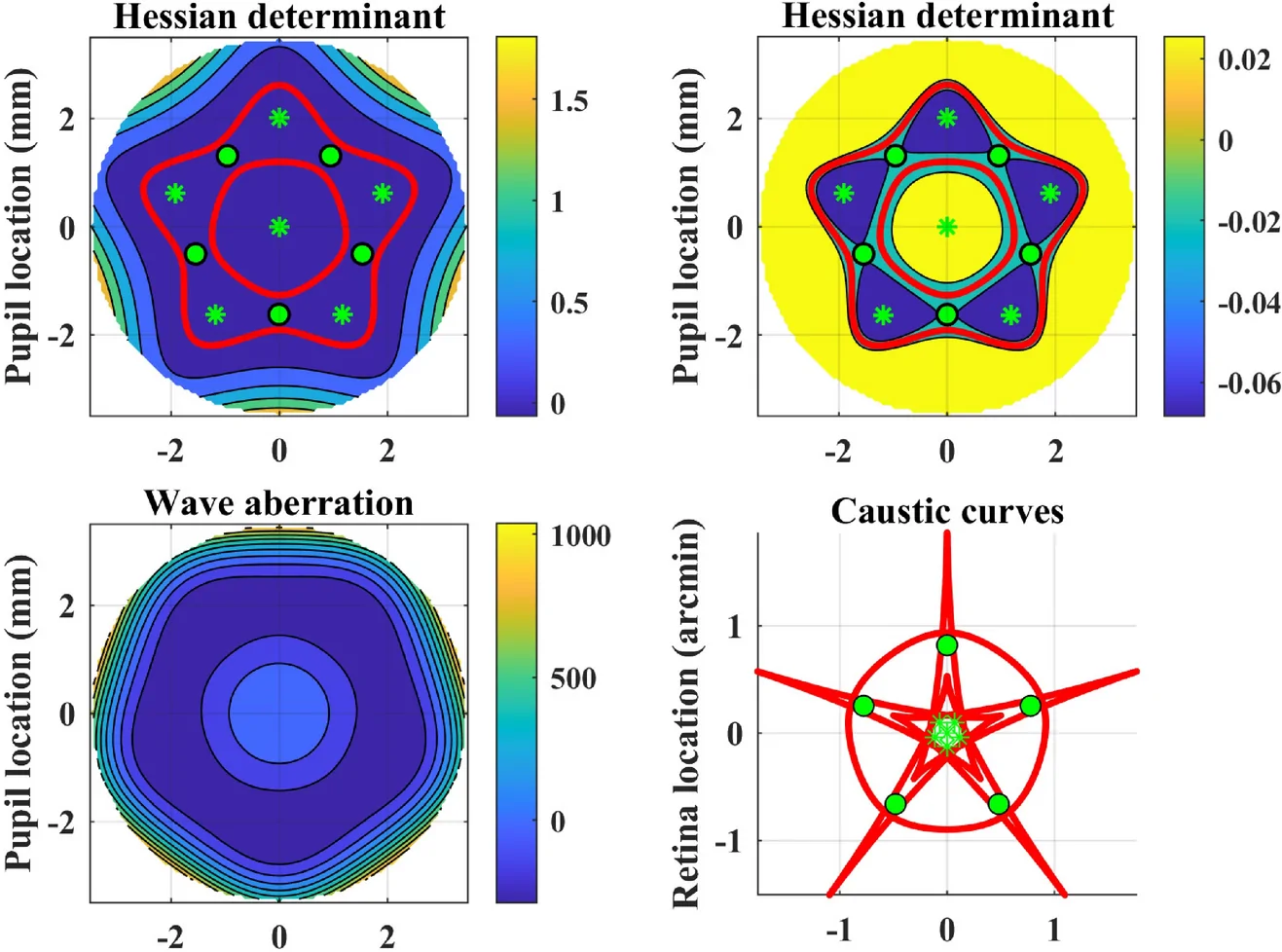
Wave aberration $W = 0.2 Z_{2}^{0} + 0.2 Z_{4}^{0} + 0.07 Z_{5}^{5}$ given in (7), Hessian determinant map, and caustic patterns of equally-distanced five-point starburst. The same Hessian map is depicted in the upper panels. However, in the right panel the colorbar is clipped to specific values around zero to reveal the fine structure of the Hessian determinant in regions located close to the cusps of Gauss. Green stars denote the cusps of Gauss, and green points surrounded by black circles denote the saddle cusps of Gauss in both the Hessian determinant map and its projection onto the retina plane.

The second example is a wave aberration given by:

7 $$\begin{aligned} W = 0.2 Z_{2}^{0} + 0.2 Z_{4}^{0} + 0.07 Z_{5}^{5}, \end{aligned}$$

which generates a 5-fold symmetry. There are 11 cusps of Gauss, five of them being fertile points (as shown in Fig. 8). In this case, each fertile cusp of Gauss generates a cusp caustic, and the action of two opposite fertile points, two converging fold caustics; the combination of both generates a long-bright starbust point. Overall, the caustic exhibits a five-point starburst pattern.

In both previous examples, Figs. 7 and 8, the number of starburst peaks equals the number of fertile cusps of Gauss, being equally-distanced odd starbursts.

#### Even equally-distanced, and non-equal-distanced peaks starbursts

A first example will be a four equally-distanced starburst obtained by a wave aberration given by:

8 $$\begin{aligned} W = 0.2 Z_{4}^{0} + 0.15 Z_{4}^{4}, \end{aligned}$$

**Fig. 9:**
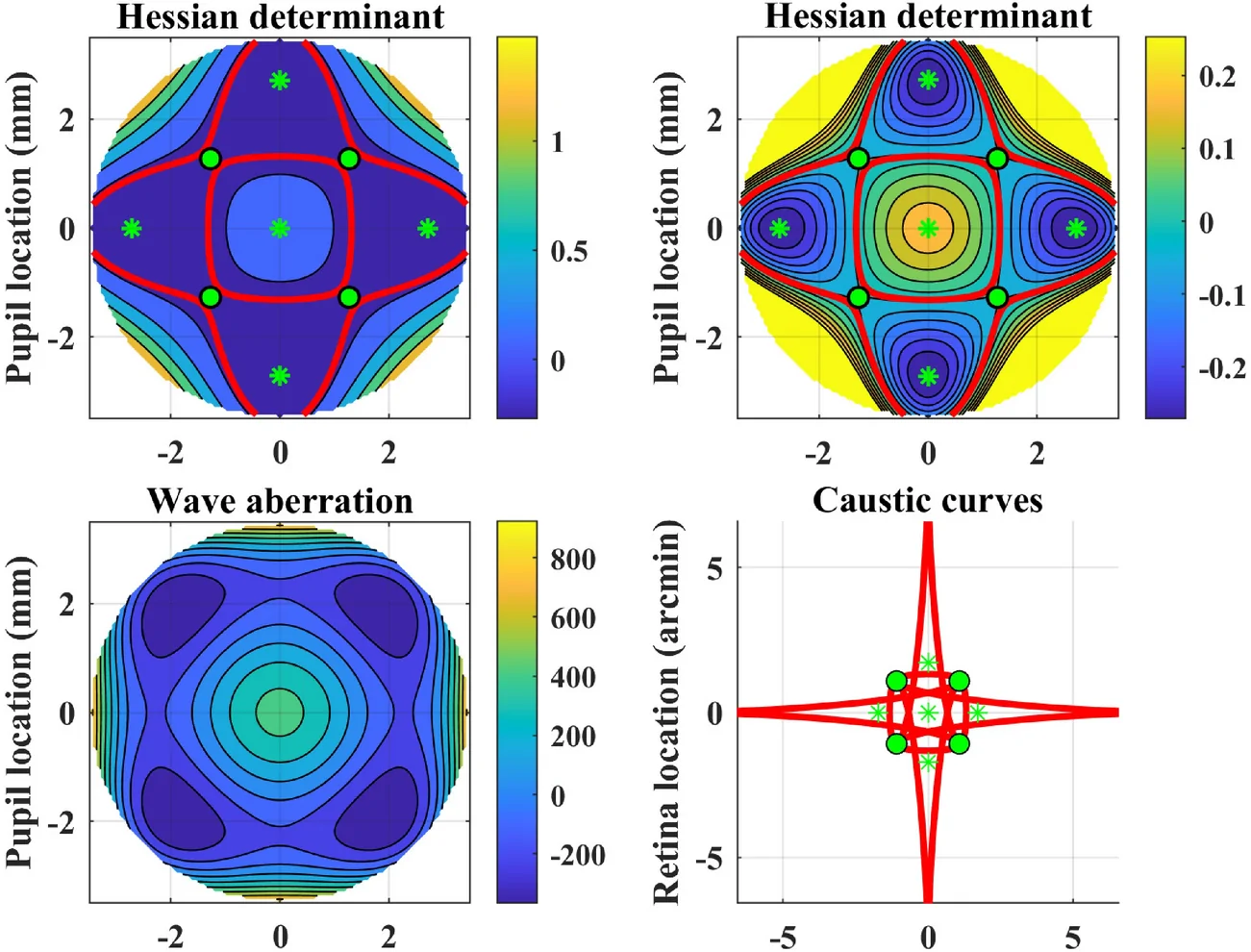
Wave aberration $W = 0.2 Z_{4}^{0} + 0.15 Z_{4}^{4}$ given in (8), Hessian determinant map, and caustic patterns of an equally-distanced four-point starburst. The same Hessian map is depicted in the upper panels. However, in the right panel the colorbar is clipped to specific values around zero to reveal the fine structure of the Hessian determinant in regions located close to the cusps of Gauss. Green stars denote the cusps of Gauss, and green points surrounded by black circles denote the saddle cusps of Gauss in both the Hessian determinant map and its projection onto the retina plane.

As in the previous subsection, Fig. 9 shows the wave aberration, the Hessian determinant, and the caustic pattern of equation (8).

There are nine cusps of Gauss, 4 of which are saddles, determining a 4-fold caustic pattern symmetry. The cusps are arranged in pairs (alternating saddle and no-saddle pairs) along lines of reflectional symmetry. Whereas the lines including the fertile cusp of Gauss induce cusp caustics, they do not generate starburst peaks because they are inside the central core; the lines including the ordinary cusp of Gauss induce peaks obtained from the convergence of two-fold caustics (generated by opposite pairs of fertile cusps of Gauss). Overall, we get an equally distanced 4-point starburst.

The second example is a six equally-distanced starburst provided by a wave aberration given by:

9 $$\begin{aligned} W = 0.2 Z_{4}^{0} + 0.19 Z_{6}^{6}, \end{aligned}$$

**Fig. 10:**
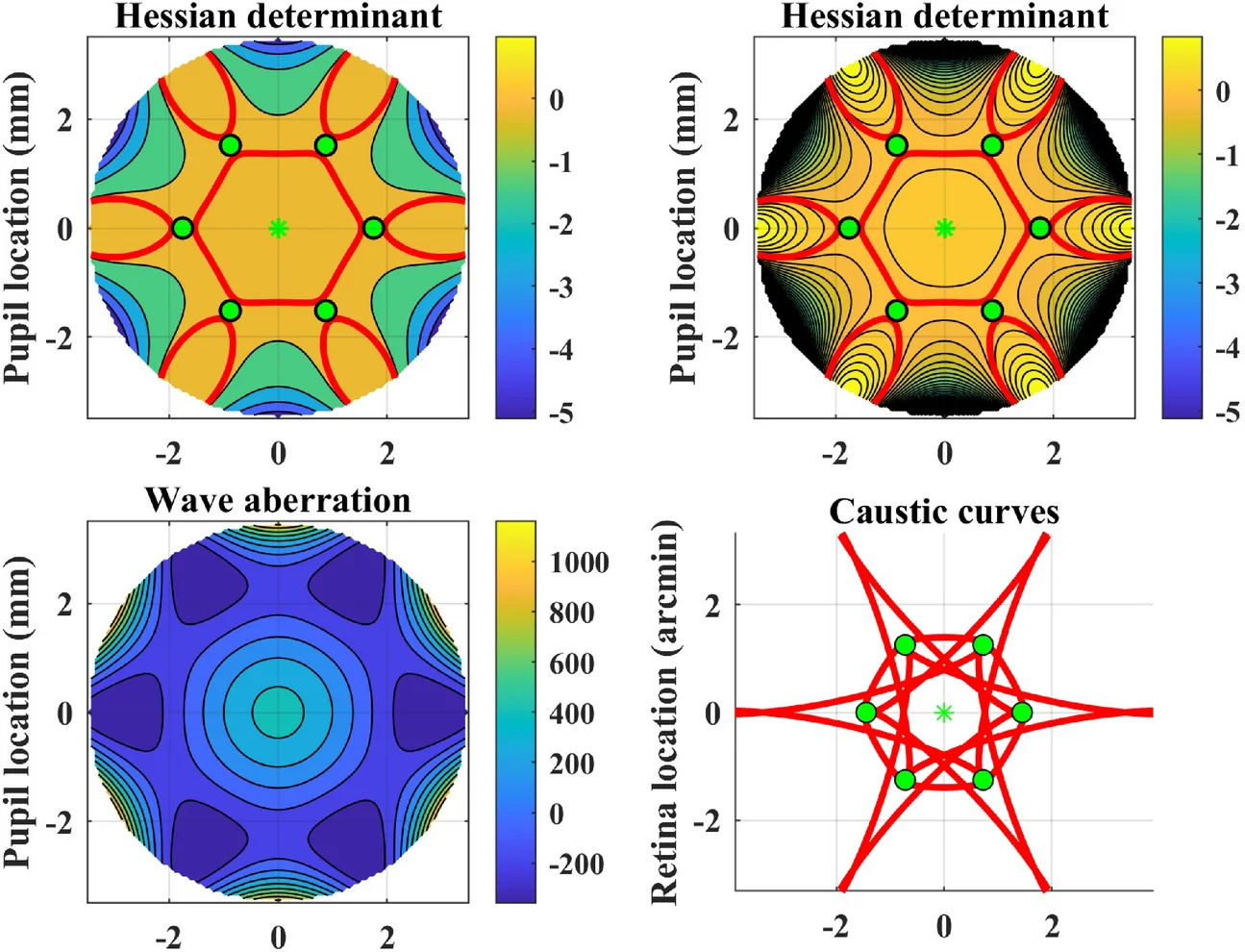
Wave aberration $W = 0.2 Z_{4}^{0} + 0.19 Z_{6}^{6}$ given in (9), Hessian determinant map, and caustic patterns of an equally-distanced six-point starburst. The same Hessian map is depicted in the upper panels. However, in the right panel the colorbar is clipped to specific values around zero to reveal the fine structure of the Hessian determinant in regions located close to the cusps of Gauss. Green stars denote the cusps of Gauss, and green points surrounded by black circles denote the saddle cusps of Gauss in both the Hessian determinant map and its projection onto the retina plane.

As in the previous subsection, Fig. 10 shows the wave aberration, the Hessian determinant, and the caustic pattern of (9).

Now, the number of cusps of Gauss is 7, 6 of which are fertile, configuring 6 lines of reflectional symmetry that determine a 6-fold caustic pattern symmetry. As in the case of the 5-point staburst, each fertile cusp of Gauss generates a cusp caustic, and the action of two opposite fertile points, two converging fold caustics; the combination of both generates a long-bright starbust point. Thus inducing an equally distant 6-point staburst.

The final example is a wave aberration generated by:

10 $$\begin{aligned} W = 0.2 Z_{4}^{0} + 0.09 Z_{4}^{4}, \end{aligned}$$

**Fig. 11:**
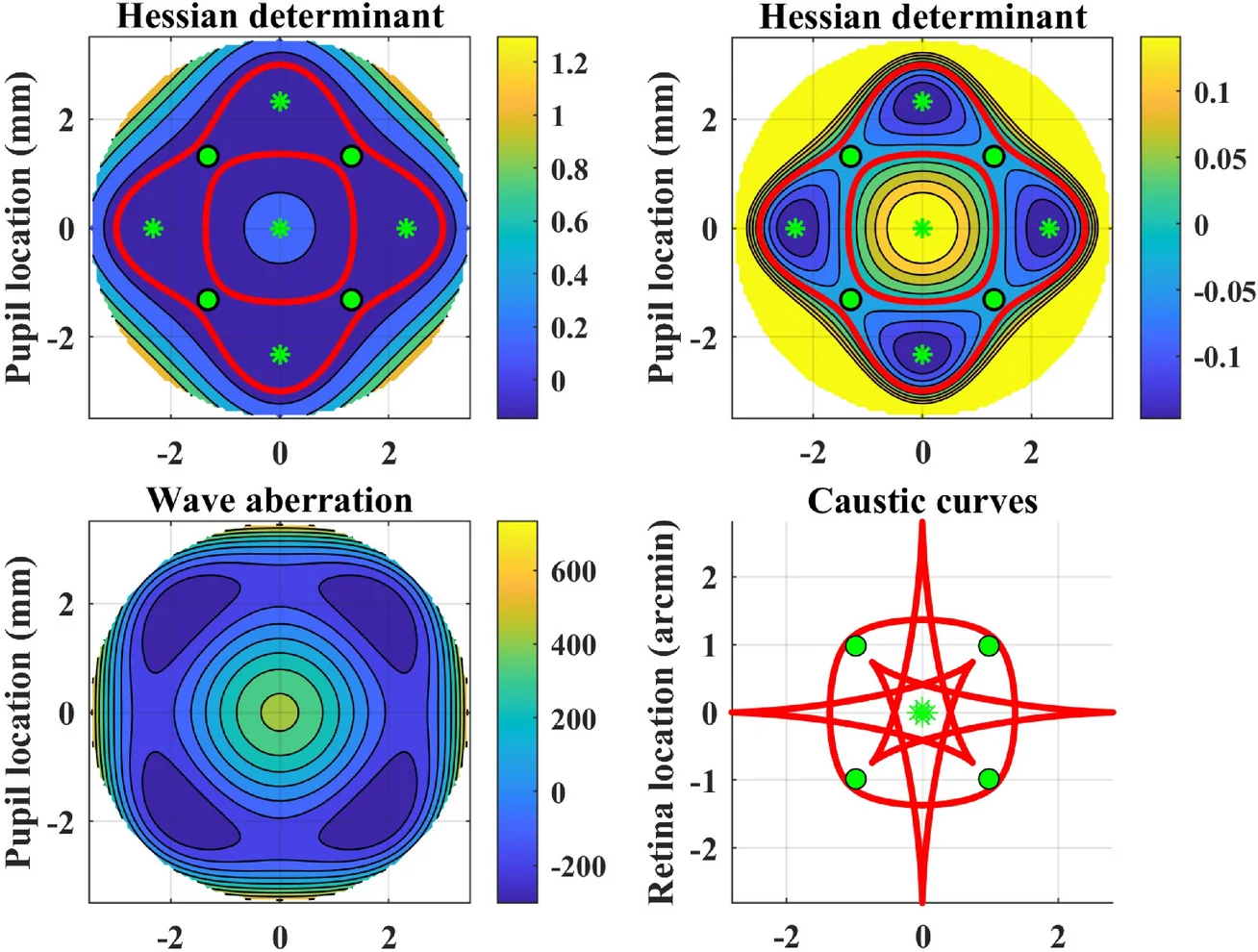
Wave aberration $W = 0.2 Z_{4}^{0} + 0.09 Z_{4}^{4}$ given in (10), Hessian determinant map, and caustic patterns of non-equal-distance eight-point starburst. The same Hessian map is depicted in the upper panels. However, in the right panel the colorbar is clipped to specific values around zero to reveal the fine structure of the Hessian determinant in regions located close to the cusps of Gauss. Green stars denote the cusps of Gauss, and green points surrounded by black circles denote the saddle cusps of Gauss in both the Hessian determinant maps and their projection onto the retina plane.

As in the case of wave aberration (8), the number of total and saddle cusps of Gauss is 9 and 4, respectively. Also, the cusps are arranged in pairs (alternating saddle and no-saddle pairs) along lines of reflectional symmetry. However, now the lines including the fertile cusp of Gauss induce short starburst peaks due to the presence of cusp caustics, and the lines including the ordinary cusp of Gauss induce long peaks obtained from the convergence of two-fold caustics (generated by opposite pairs of fertile cusps of Gauss). Then, the structure of caustic lines (see Fig. 11) creates not a 4-point starburst but an 8-point starburst, where 4 peaks are longer than the other 4. Hence, this exemplifies a non-equal-distance starburst as stated in Corollary 6.

## Discussion

Our theory enables us to predict whether someone could see a star-type starburst pattern knowing his/her subject’s wave aberration, and, in that case, to predict an upper bound to the number of starburst peaks seen. For this to happen, a certain combination of symmetric aberrations and a $Z_n^n$ term –being dominant over the rest of the Zernike monomials– must exist. We note that, in some cases, there are two caustic cusps associated with each point (see Figs. 7, 8, and 10), which presumably would induce a brighter sensation of peaks than in other cases.

Although we have provided the necessary conditions to perceive those types of starbursts, we warn that they may not be sufficient. Besides excessive blurring, scattering from artificial light (e.g., streetlights) may also prevent seeing starburst because of insufficient luminance contrast^1^. This scattering may be induced either in the atmosphere or within the eye, which occurs when the retina receives light directly from artificial light sources, producing visual skyglow^27^. There is still another reason for perceiving a *star*: thin light lines radiating from the central bright spot may be formed by edge diffraction produced by fine structures within the human eye. These fine structures could, under special viewing conditions, interfere with vision, such as squinting eyelids that block the pupil. They can also be generated by permanent ocular conditions such as cataracts, lens Y-suture, or corneal swelling. In passing, it’s worth mentioning that edge diffraction induced by mirror spiders causes the diffraction spikes found in images registered by reflective telescopes^28,29^.

We also provided threshold defocus values to avoid destroying the star-like starburst. On the one hand, the defocus may produce a much more complex caustic pattern. For instance, a snowflake pattern (as shown in^3^ specifically in Fig. 2) may occur when the saddle cusps of Gauss separate from the pupil center; on the other hand, the defocus may create excessively blurred patterns, even to discern a hot spot in the energy. The sensitivity of starburst patterns to defocus has also been analyzed theoretically in^2^. Last but not least, if the retina starburst extension is too small, its shape may not be resolved simply due to the human eye’s visual resolution.

Conversely, knowing how a subject sees a star (*p*-fold symmetry and the number of peaks), a promising application of our theoretical framework is that we could infer some basic properties of his/her wave aberration function. Recovering some properties of the wave aberration function from the caustic pattern is an unexplored and exciting problem, somewhat mathematically connected to the so-called Hessian topology^30^. This problem was recently addressed by Rubinstein^31^. This problem has far-reaching potential applications in multifocal optical design^32^, wavefront sensing^33^, or even in electron optics^34,35^.

Finally, we would like to mention another possible application. When a light beam propagates through a medium, it diffuses due to diffraction, causing the transverse intensity patterns to deviate significantly from the original plane. A major goal in current optical engineering is to obtain beams where these deviations are considerably reduced, the so-called nondiffracting beams. Recently, new non-diffracting polygonal beams, with p-fold symmetry, have been proposed^36,37^. In these beams, the number of degrees of freedom (parameters controlling the beam shape) also determines the type of symmetries^38^. The intensity patterns can be described by a set of curved caustic lines converging onto intensity cusps that form the vertices of a regular polygon, thus showing patterns very similar to starbursts. Therefore, we believe that our symmetry-preserving p-fold theoretical formalism is directly connected to polygon light beams, so some theoretical feedback between these research fields could be established.

## Data Availability

No datasets were generated or analysed during the current study.

## Proof of Proposition 4

Without loss of generality, we consider $C_{4,0}>0$. We perform the partial derivatives of *G* for *x* and *y*, and after a change of variable to polar coordinates $(x,y)=(\rho \sin \theta , \rho \cos \theta )$, and taking into account that $\cos (n\theta )=T_{n}(\cos (\theta ))$ and $\sin (n\theta )=\sin (\theta )U_{n-1}(\cos (\theta ))$, being $T_n$ and $U_n$ the Chebyshev polynomials of the first and second kind, respectively, we obtain the equations

11 $$\begin{aligned}&\rho \sin (\theta ) \Big ( A(\rho ) + B(\rho ) U_{n-2}(\cos (\theta )) \Big ) = 0, \end{aligned}$$

12 $$\begin{aligned}&\rho \Big ( A(\rho ) \cos (\theta ) - B(\rho ) T_{n-1}(\cos (\theta )) \Big ) = 0, \end{aligned}$$

where

13 $$\begin{aligned} A(\rho )= 192 \,C_{4,0}(\sqrt{15} \,C_{2,0} -15 \,C_{4,0}) +8640 \,C_{4,0}^2 \rho ^2 -C_{n,n} ^2 (n-2) (n-1)^2 n^2 (n+1) \rho ^{2 n-6} \end{aligned}$$

and

14 $$\begin{aligned} B(\rho )=12 \sqrt{10} \,C_{4,0} \,C_{n,n} (n-1) n^2 \sqrt{n+1} \rho ^{n-2}. \end{aligned}$$

Then, looking at the first equation (11), we need to study three different situations:

1. *ρ =0*.
2. $$\rho \ne 0$$ and $$\sin (\theta )=0$$.
3. $$\rho \ne 0$$, $$\sin (\theta )\ne 0$$ and $$A(\rho ) + B(\rho ) U_{n-2}(\cos (\theta ))=0$$.

Let us study each case.

**Case** *ρ =0*. In this case, the unique critical point is located at the center of the pupil, but it is never a saddle point.

**Case** $\rho \ne 0, \, \sin (\theta )=0$. This situation, corresponds with either *θ =0* or $\theta =\pi$.

Taking $\boxed {\theta =0}$, and assuming $\rho \ne 0$, equation (12) becomes $A(\rho ) - B(\rho )=0.$

Suppose that $(\rho ^*,0)$ is a solution of (11) and (12) with $\rho ^*\ne 0$, which means that

15 $$\begin{aligned} A(\rho ^*) - B(\rho ^*)=0. \end{aligned}$$

Then, for $(\rho ^*,\frac{2k\pi }{n})$, *k=1,… , n-1*, equations (11) and (12) simplify to

16 $$\begin{aligned}&\sin \Big (\frac{2k\pi }{n}\Big ) \Big ( A(\rho ^*)+B(\rho ^*) U_{n-2}\Big (\cos \Big (\frac{2k\pi }{n}\Big )\Big ) \Big )=0, \end{aligned}$$

17 $$\begin{aligned}&A(\rho ^*)\cos \Big (\frac{2k\pi }{n}\Big )-B(\rho ^*) T_{n-1}\Big (\cos \Big (\frac{2k\pi }{n}\Big )\Big ) =0. \end{aligned}$$

In the case *k≠ n/2*, equations (16) and (17) reduce to

18 $$\begin{aligned}&A(\rho ^*)-B(\rho ^*) =0, \nonumber \\&\big ( A(\rho ^*)-B(\rho ^*) \big ) \cos \Big (\frac{2k\pi }{n}\Big ) =0, \end{aligned}$$

which hold by (15).

In the case *k=n/2*, equation (17) also becomes as (18), and (16) vanishes since $\sin \big (\frac{2k\pi }{n}\big )=0$.

With this, we have proved that if $(\rho ^*,0)$ is a critical point, then $(\rho ^*,\frac{2k\pi }{n})$ for *k=0,...,n-1* are also critical points.

For the case $\boxed {\theta =\pi }$, the equations (11) and (12) for computing the critical points reduce to

$$\begin{aligned} A(\rho )+(-1)^{n-1} B(\rho )=0. \end{aligned}$$

An analog argument, as in the previous case, leads us to the same conclusion: if $(\rho ^*,\pi )$ is a critical point, then $(\rho ^*,\pi +\frac{2k\pi }{n})$ for *k=0,...,n-1* are also critical points.

**Case** $\rho \ne 0, \, \sin (\theta )\ne 0$. Now, equations (11) and (12) for computing the critical points simplify to:

$$\begin{aligned} & A(\rho ) + B(\rho ) U_{n-2}(\cos (\theta )) = 0, \\ & A(\rho ) \cos (\theta ) - B(\rho ) T_{n-1}(\cos (\theta )) = 0. \end{aligned}$$

Then, taking apart *A(ρ )* in the first equation and substituting it in the second, one gets

20 $$\begin{aligned} & A(\rho ) =- B(\rho ) U_{n-2}(\cos (\theta )), \\ & B(\rho )\big ( U_{n-2}(\cos (\theta )) \cos (\theta ) + T_{n-1}(\cos (\theta ))\big ) = 0. \end{aligned}$$

As $B(\rho )\ne 0$, equation (20) reduces to $\sin (n\theta )=0$, using the definition of Chebyshev polynomials. Then, $\cos (n\theta )=\pm 1$, depending on whether $n\theta =2k\pi$ or $n\theta =(2k+1)\pi$, with $k\in \mathbb {Z}$.

With this, we finally have

$$\begin{aligned} A(\rho ) - B(\rho )= 0 \text { if } n\theta =2k\pi , \end{aligned}$$

or

$$\begin{aligned} A(\rho ) + B(\rho )= 0 \text { if } n\theta =(2k+1)\pi . \end{aligned}$$

Moreover, one can prove that when $\theta =(2k+1)\pi /n$, *k=0,… ,n-1*, then the Hessian determinant of *G* is always the same; therefore, all the corresponding critical points are saddle points or not. The same situation occurs in the case $\theta =2k\pi /n$.

## References

[1] Bará, S., Falchi, F., Lima, R. C. & Pawley, M. Can we illuminate our cities and (still) see the stars?. *Int. J. Sustain. Light.* **23**, 58–69 (2021).

[2] Rubinstein, J. On the geometry of visual starbursts. *J. Opt. Soc. Am. A* **36**, B58–B64 (2019). 10.1364/JOSAA.36.000B58 31044956

[3] Xu, R., Thibos, L., Lopez-Gil, N., Kollbaum, P. & Bradley, A. Psychophysical study of the optical origin of starbursts. *J. Opt. Soc. Am. A* **36**, B97–B102 (2019). 10.1364/JOSAA.36.000B97 31044967

[4] Stavroudis, O. N. The optics of rays, wavefront, and caustics (Academic Press, 1972).

[5] Navarro, R. & Losada, M. A. Shape of stars and optical quality of the human eye. *J. Opt. Soc. Am. A* **14**, 353–359 (1997). 10.1364/josaa.14.000353 9014354

[6] Atchison, D. & Smith, G. *Optics of the human eye* 2nd edn. (CRC Press, 2023).

[7] Barbero, S., Bradley, A., Lopez-Gil, N., Rubinstein, J. & Thibos, L. Catastrophe optics theory unveils the localised wave aberration features that generate ghost images. *Ophthalmic. Physiol. Opt.* **42**, 1074–1091 (2022). 35620968 10.1111/opo.13008 PMC9543491

[8] Niu, K. & Tian, C. Zernike polynomials and their applications. *J. Opt.* **24**, 123001 (2022).

[9] Zernike, F. & Stratton, F. Diffraction theory of the knife-edge test and its improved form, the phase-contrast method. *Mon. Not. R. Astron. Soc.* **94**, 377–384. 10.1093/mnras/94.5.377 (1934).

[10] Dunkl, C. F. & Xu, Y. Orthogonal Polynomials of Several Variables. Encyclopedia of Mathematics and its Applications, 2 edn (Cambridge University Press, 2014).

[11] Born, M. & Wolf, E. *Principles of optics: Electromagnetic theory of propagation, interference and diffraction of light* (Pergamon Press, 1965).

[12] Lakshminarayanan, V. & Fleck, A. Zernike polynomials: A guide. *J. Mod. Opt.* **58**, 1678–1678. 10.1080/09500340.2011.633763 (2011).

[13] Szegö, G. *Orthogonal Polynomials* (American Mathematical Society, 1975).

[14] Nye, J. *Natural focusing and fine structure of light: caustics and wave dislocations* (Institute of Physics Publishing, 1999).

[15] Berry, M. & Upstill, C. IV. Catastrophe optics: Morphologies of caustics and their diffraction patterns. In *Progress in Optics 18* 257–346 (Elsevier, 1980). 10.1016/S0079-6638(08)70215-4.

[16] Montaldi, J. *Singularities, bifurcations and catastrophes* (Cambridge University Press, 2021).

[17] Porteous, I. Geometric differentiation. For the Intelligence of curves and surfaces (Cambridge University Press, 2001).

[18] Blaker, J. W. & Tavel, M. A. The application of Noether’s theorem to optical system. *Am. J. Phys.* **42**, 857–861 (1974).

[19] Buchdahl, H. A. *An introduction to Hamiltonian optics* (Dover Publications, 1993).

[20] Bruce, J. W. & Tari, F. Extrema of principal curvature and symmetry. *Proc. Edinb. Math. Soc.* **39**, 397–402 (1996).

[21] Kreyszig, E. *Differential geometry* (Dover, 1991).

[22] Torre, A. *Linear ray and wave optics in phase space: bridging ray and wave optics via the Wigner phase-space picture* (Elsevier, 2005).

[23] Qi, L. Extrema of a real polynomial. *J. Glob. Optim.* **30**, 405–433 (2004).

[24] Durfee, A., Munson, N. K. H., Roy, J. & Westby, I. Counting critical points of real polynomials in two variables. *Am. Math. Mon.* **100**, 255–271 (1993).

[25] Thibos, L., Bradley, A. & Hong, X. A statistical model of the aberration structure of normal, well-corrected eyes. *Ophthalmic. Physiol. Opt.* **22**, 427–433 (2002). 12358314 10.1046/j.1475-1313.2002.00059.x

[26] Kingston, A. & Cox, I. Population spherical aberration: Associations with ametropia, age, corneal curvature, and image quality. *Clin Ophthalmol* **7**, 933–938 (2013). 23723685 10.2147/OPTH.S44056 PMC3666881

[27] Bará, S. & Bao-Varela, C. Skyglow inside your eyes: Intraocular scattering and artificial brightness of the night sky. *Int. J. Sustain. Light.* **25**, 1–9 (2023).

[28] Harvey, J. E. & Ftaclas, C. Diffraction effects of telescope secondary mirror spiders on various image-quality criteria. *Appl. Opt.* **34**, 6337–6349 (1995). 21060478 10.1364/AO.34.006337

[29] Lendermann, M., Tan, J. S. Q., Koh, J. M. & Cheong, K. H. Computational imaging prediction of starburst-effect diffraction spikes. *Sci. Rep.* **8**, 16919 (2018). 30446668 10.1038/s41598-018-34400-z PMC6240111

[30] Arnold, V. *Arnold’s Problems* (Springer-Verlag, 2004).

[31] Rubinstein, J. Forward and inverse problems for symmetric starbursts. *J. Opt. Soc. Am. A* **3**, 430–439 (2026). 10.1364/JOSAA.582961 41842567

[32] Barbero, S. Multifocal wavefronts with prescribed caustics in axially symmetric optical systems. *Opt. Express* **30**, 14274–14286 (2022). 35473174 10.1364/OE.453322

[33] Ribak, E. N. Harnessing caustics for wave-front sensing. *Opt. Lett.* **26**, 1834–1836 (2001). 18059709 10.1364/ol.26.001834

[34] Fraysse, T., Cours, R., Lourenço-Martins, H. & Houdellier, F. Morphologies of caustics studied by catastrophe charged-particle optics. arXiv Preprint arxiv:2509.09551. (2025). 10.1016/j.ultramic.2025.114291 41519016

[35] Tavabi, A. H., Migunov, V., Dwyer, C., Dunin-Borkowski, R. E. & Pozzi, G. Tunable caustic phenomena in electron wavefields. *Ultramicroscopy* **157**, 57–64. 10.1016/j.ultramic.2015.04.003 (2015). 26069930

[36] Barwick, S. Accelerating regular polygon beams. *Opt. Lett.* **35**, 4118–4120. 10.1364/OL.35.004118 (2010). 21165109

[37] Zhong, W.-P., Zhong, W., Belić, M. & Yang, Z. Controllable two-dimensional diffraction-free polygon beams. *Phys. Lett. A* **432**, 128009. 10.1016/j.physleta.2022.128009 (2022).

[38] Yang, Z., Zhong, W.-P. & Belić, M. Modal structure of two-dimensional nondiffracting Weber beams. *Phys. Lett. A* **560**, 130955. 10.1016/j.physleta.2025.130955 (2025).

